# Surveillance for Avian Influenza in Wild Birds in the Lombardy Region (Italy) in the Period 2022–2024

**DOI:** 10.3390/v16111668

**Published:** 2024-10-24

**Authors:** Tiziana Trogu, Silvia Bellini, Sabrina Canziani, Maya Carrera, Chiara Chiapponi, Mario Chiari, Marco Farioli, Alice Fusaro, Enrico Savegnago, Ambra Nucci, Laura Soliani, Alessio Bortolami, Antonio Lavazza, Calogero Terregino, Ana Moreno

**Affiliations:** 1 Istituto Zooprofilattico Sperimentale della Lombardia e dell’Emilia Romagna “Bruno Ubertini” (IZSLER), Via Antonio Bianchi 7/9, 25124 Brescia, Italy; tiziana.trogu@izsler.it (T.T.); silvia.bellini@izsler.it (S.B.); sabrina.canziani@izsler.it (S.C.); maya.carrera@izsler.it (M.C.); chiara.chiapponi@izsler.it (C.C.); ambra.nucci@izsler.it (A.N.); laura.soliani@izsler.it (L.S.); antonio.lavazza@izsler.it (A.L.); 2 Direzione Generale Welfare, U.O. Veterinaria, Piazza Città di Lombardia 1, 20124 Milano, Italy; mario_chiari@regione.lombardia.it (M.C.); marco_farioli@regione.lombardia.it (M.F.); 3 Istituto Zooprofilattico Sperimentale delle Venezie, Viale dell’Università 10, 35020 Padova, Italy; afusaro@izsvenezie.it (A.F.); esavegnago@izsvenezie.it (E.S.); abortolami@izsvenezie.it (A.B.); cterregino@izsvenezie.it (C.T.)

**Keywords:** avian influenza, wild birds, Italy, low pathogenic avian influenza (LPAI) viruses, H5N1 HPAIV clade 2.3.4.4b

## Abstract

Influenza A virus (AIV) circulation was investigated in the Lombardy region, during 2022–2024, in wild ducks (through hunting and sampling of faecal samples within natural parks) and wild birds found dead. Samples were analysed through real-time RT-PCRs for Influenza A virus, H5 and H7. Whole genome sequencing was performed on AIV-positive samples. Screening of 3497 hunted Anatidae revealed a total of 184 positive samples. Complete sequencing of 136 samples highlighted the presence of 21 different subtypes ranging from H1N1 to H12N5. The H5N1 HPAIV (high pathogenic AIV) subtype, clade 2.3.4.4b, was the most common during the 2022–2023 winter season (31.8%), while H5 LPAI (low pathogenic AIV) strains were the most prevalent (28.6%) in the 2023–2024 season. The molecular survey on wild birds found dead (*n* = 481) showed two positive buzzards (14%, 2/14), one grey heron (5.5%, 1/18) and one kestrel (7.6%, 1/13). Regarding the order of *Charadriiformes*, the dead gulls sampled in 2022 (17 birds) were all negative, whereas 85 out of 167 (51%) individuals were positive in 2023. All positives were caused by an H5N1 HPAIV clade 2.3.4.4b virus belonging to genotype BB. All the faecal samples (1699) received from passive surveillance in nature parks were analysed for AIV with negative results.

## 1. Introduction

Avian influenza viruses (AIV) are able to spread and replicate within a wide range of animal species, thus hampering global disease eradication efforts [1,2]. Each year, various strains of these viruses circulate widely among wild bird species, which act as their natural reservoirs and are capable of transmitting the viruses to other domestic and wild birds and accidental hosts, including aquatic and terrestrial mammals and humans [3,4,5]. During migratory seasons, AIV can spread to new territories and regions, even considerably distant ones, posing a significant threat to public and animal health. Wild waterfowl belonging to the orders *Anseriformes* and *Charadriiformes* are important reservoirs of low pathogenic (LPAIV) but also high pathogenic (HPAIV) avian influenza viruses, which can be transmitted directly or indirectly to domestic birds [6]. The orofecal transmission route of most LPAI viruses and their typically low virulence in waterfowl create the ideal conditions for its dissemination [7]. The variability of circulating subtypes in *Anatidae* species [8] and the rapid population turnover of many duck species favour co-infections, facilitating genetic reassortment with the subsequent emergence of new genotypes [9].

To understand the epidemiological evolution of avian influenza and its increasing spread, it is necessary to start from the wave of HPAI outbreaks, caused by H5 viruses derived from the A/Goose/Guangdong/1/1996 (Gs-Gd), that caused severe economic losses to the poultry industry in many countries. This strain originated in southeast Asia in 1996, becoming endemic in several countries of the region since 2003 [10]. The H5 HPAI viruses, previously largely confined to poultry, were shown to be able to infect a broad range of avian and mammalian species, including humans [11]. In the first phases of circulation, haemagglutinin (HA) genes have undergone some variations creating different lineages. Still, since 2009, several HPAI subtypes have been the subject of increased reassortment phenomena between GsGd and other low-pathogenic influenza viruses, which then resulted in the formation of HPAI H5 viruses, clade 2.3.4.4 increasing the incidence of the disease worldwide [5,12,13]. Within this clade, two genetic groups (A and C) were detected in China and South Korea in late 2013 [11]. The clade 2.3.4.4c spread to Europe in 2014–2015 [14], giving way to clade 2.3.4.4.b in late October 2016, which was isolated from Hungarian poultry and ducks in Germany [15] and then spread throughout Europe [11]. In particular, the H5 HPAIV viruses of clade 2.3.4.4b are highly prone to reassort with other Influenza A viruses present in birds, leading to the generation of a large number of different subtypes and genotypes, such as H5N1 [16], H5N2 [17], H5N3 [18], H5N4 [19], H5N5 [20], H5N6 [21] and H5N8 [22], found in both domestic and wild birds in Europe over the past decade. These viruses have demonstrated a marked ability to undergo species jump and to infect a wide range of domestic and wild mammals, including humans [3,4,5]. Severe systemic infections, increased neurotropism and high mortality rates were detected among wild birds and mammals [23,24,25]. Recently, infections caused by viruses of this clade were first reported in several dairy cattle farms in the United States [26]. Considering the scale of the currently ongoing outbreaks, the increased chances for virus adaptation and the proven zoonotic potential, infection in cows poses a great risk to human health through direct contact with animals and ingestion of raw milk.

In such a context, in which the protagonist is a plastic and versatile virus, able to adapt to multiple hosts and spread to very distant and different geographical areas, it is essential to set up surveillance systems to monitor the epidemiological evolution of this disease at regional and national levels. Surveillance should be developed and conducted in an integrated system that includes not only domestic and wild birds and mammals but also the environment. The aim of such a surveillance system is the early detection of circulating Influenza A, H5 and H7 viruses, with a focus on HPAIV, in order to protect domestic poultry and safeguard human and animal health [11].

A national surveillance plan is in place for poultry, game bird farms and wild birds in Italy. Recently, the Lombardy region implemented its monitoring system through the sampling of wild duck faeces within protected areas, and since the end of 2020, to further increase the efficiency of monitoring in identifying viral introductions, sampling of wild birds shot during hunting was activated in several sites in high-risk areas distributed across four provinces (Brescia, Cremona, Mantua, Pavia). This study aims to report the results of active and passive surveillance of wild birds in Lombardy in the period 2022–2024 as part of the regional and national wildlife and avian influenza surveillance programmes.

## 2. Materials and Methods

### 2.1. Sample Collection

#### 2.1.1. Active Surveillance of Hunted Anatidae

**Figure 1:**
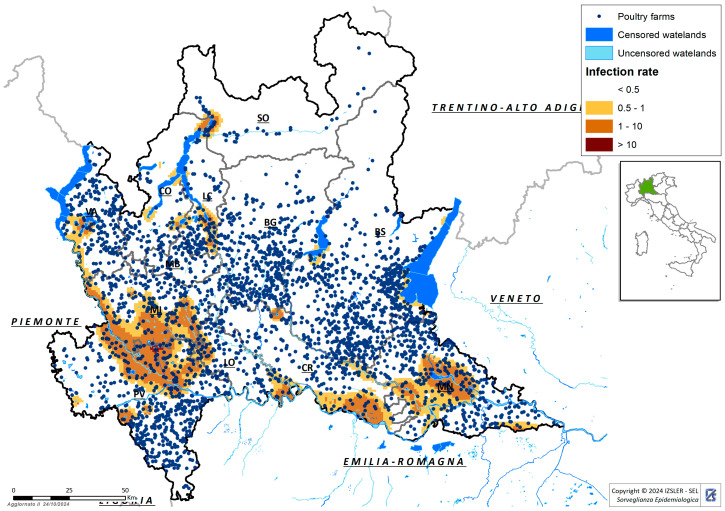
In the map, the distribution of poultry farms (black dots), the wetlands censused (dark blue) and the infection rate in the different areas of the region are shown. These factors have been considered in the risk analysis to choose the sheds’ localization.

In Lombardy, a risk analysis was conducted to identify the areas that presented a greater risk of virus circulation, and in which, sampling was then carried out. To conduct the risk analysis, the following factors were considered: (i) the georeferencing of wetlands; (ii) the census of wild Anatidae; (iii) available data on movements and resting and feeding sites of Anatidae; (iv) the density of intensive domestic poultry farms; and finally, (v) the estimated prevalence of AI. These factors allowed us to identify the zones of risk for AIV introduction and diffusion (Figure 1), corresponding to the higher density of poultry farms in areas with abundant rivers and ponds. Wetlands represent the most suitable habitat for Anatidae, becoming places of aggregation and feeding.

**Figure 2:**
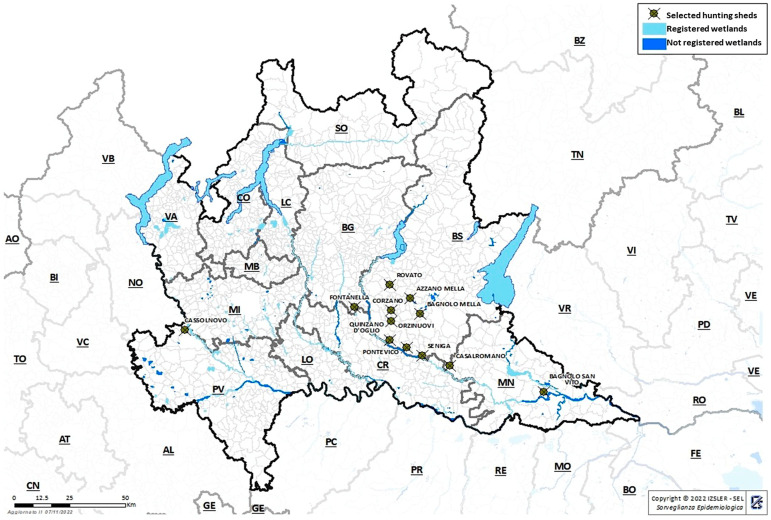
Active surveillance of hunted Anatidae. Selected hunting sheds are shown.

Once the areas at greatest risk had been identified, 12 hunting sheds were selected (shown in Figure 2). Hunters were trained to sample cloacal swabs from all the Anatidae hunted. The samples collected during two hunting seasons, 2022–2023 and 2023–2024 (from mid-September to the end of January), were placed in 1 mL of PBS with 10% glycerine added and delivered to the laboratory as soon as possible. They were analysed individually.

#### 2.1.2. Surveillance in Parks

**Figure 3:**
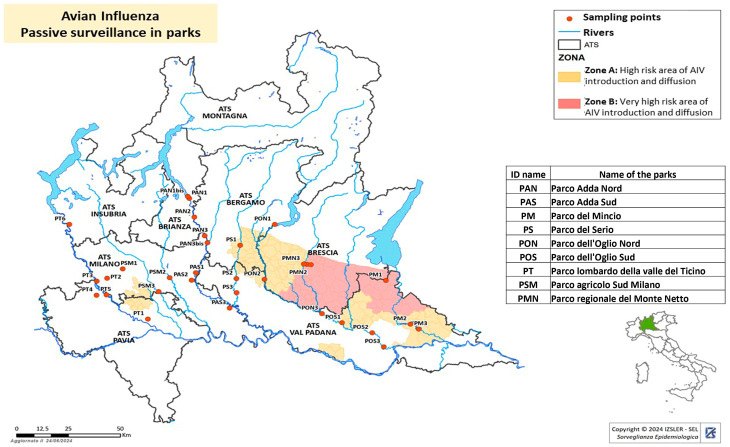
Avian influenza passive surveillance in nine regional parks. High-risk zones A and B are evidenced. Sampling points are shown with red dots. The black borders represent the boundaries of the ATS (Health Authority responsible at the territorial level).

Nine regional parks were selected (Parco Adda Nord, Parco Adda Sud, Parco del Mincio, Parco del Serio, Parco dell’Oglio Nord, Parco dell’Oglio Sud, Parco lombardo della valle del Ticino, Parco Agricolo Sud Milano and Parco regionale del Monte Netto) (Figure 3). Three sample points were identified within each park, prioritising the sites selected by ducks for breeding (e.g., ponds, ditches with water present continuously, etc.) and close to the areas with a higher density of intensive poultry farms. The identified points were georeferenced. For each sampling point, a maximum of 20 faecal samples were collected every 15 days from 2022 to 2024. Samples were analysed in pools of 5.

#### 2.1.3. Passive Surveillance on Wild Birds Found Dead on the Territory

From each bird found dead and delivered to Istituto Zooprofilattico Sperimentale della Lombardia e dell’Emilia-Romagna (IZSLER) in the period 2022–2023, a pool of tissues such as brain, lung and intestine was collected during the necropsy and then homogenised and stored for subsequent investigation of the presence of AIVs.

### 2.2. AIV Genome Detection and Whole Genome Sequencing

From all samples, viral RNA was extracted with DSP Virus/Pathogen mini kit (Qiagen, Hilden, Germany) using the QiaSymphony SP/AS (Qiagen, Hilden, Germany) according to the manufacturer’s protocol, with a final elution volume of 60 μL. The search for the AIV genome was performed using a real-time RT-PCR whose target is the M gene as described in [27]. Samples that tested positive for AIV were subsequently tested for H5 and H7 genomes through two real-time RT-PCR tests [27,28]. Samples positive for H5 or H7 were sent to the IZSVe Reference Centre for confirmation of positivity, determination of pathotype and whole genome sequencing, following the protocol previously described in Fusaro et al., 2019 [29].

For subtype assignment, whole genome sequencing of all the other AIV-positive samples was performed at IZSLER as previously described by Lycett et al. [30] using the SuperScript^®^ III One-Step RT-PCR system with Platinum^®^ Taq High Fidelity (Thermo Fisher Scientific, Waltham, Massachusetts, USA). RT-PCR products were purified with NucleoSpin^®^ Gel and PCR Clean-up (Macherey-Nagel, Carlo Erba, Milan, Italy). DNA libraries were prepared with the NEX-TERA-XT DNA Library Prep kit (Illumina Inc. San Diego, California, USA) according to the manufacturer’s instructions. Pooled libraries were sequenced on a MiSeq instrument (Illumina Inc. San Diego, California, USA) using a Miseq Reagent Nano Kit v2 (Illumina Inc. San Diego, California, USA) in a 2 × 150 bp paired-end run. Data were assembled de novo with the CLC genomic workbench v.11 (Qiagen, Milan, Italy). Gene sequences were then aligned with ClustalW using MEGAX. Phylogenetic trees were inferred using the maximum likelihood (ML) method implemented in IQ-TREE (v 2.3.6) for the H5 viruses. The robustness of the ML trees was assessed statistically by bootstrap analysis with 1000 bootstrap samples HPAI H5N1 genotypes were assessed as described by Fusaro et al., 2024 [31].

## 3. Results

### 3.1. Active Surveillance of Hunted Anatidae

**Figure 4:**
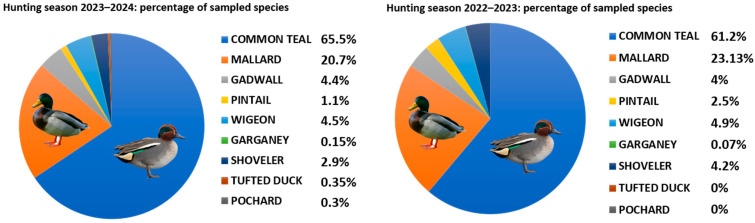
Percentage of sampled species in the hunting seasons 2022–2023 and 2023–2024.

A total of 3497 cloacal swabs were collected and analysed from hunted Anatidae; in particular, 1504 swabs were sampled during the hunting season 2022–2023 and 1993 in 2023–2024. The two most frequently sampled species were Eurasian/common teal (*Anas crecca*), with a percentage of 61,2% in 2022–2023 and 65.6% in 2023–2024, and mallard (*Anas platyrhynchos*) with 23.13% in 2022–2023 and 20.7% in 2023–2024. The other species sampled were shoveler (*Spatula clypeata*), wigeon (*Anas Penelope*), pintail (*Anas acuta*), gadwall (*Mareca strepera*), garganey (*Spatula querquedula*), pochard (*Aythya farina*) and tufted duck (*Aythya fuligula*) but in a marked lower percentage, around 15.67% in 2022–2023 and 13.7% in 2023 (Figure 4).

**Figure 5:**
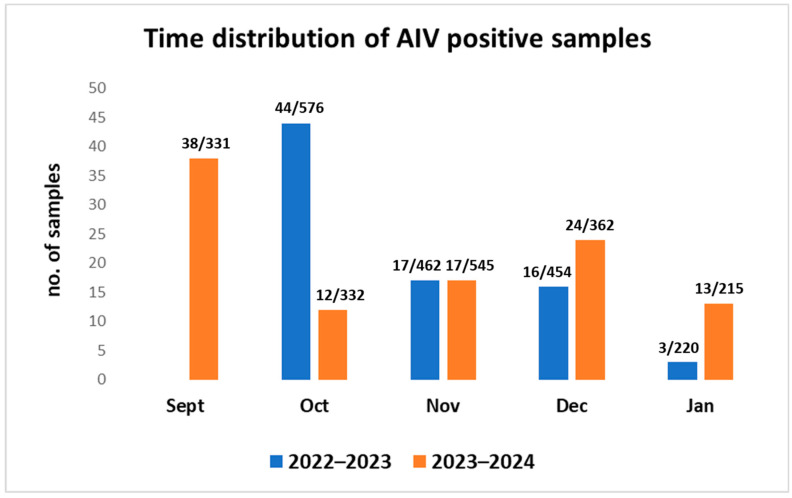
Time distribution of hunted ducks found positive for Influenza A viruses.

The screening of all cloacal swabs revealed a total positivity rate for AIVs of 5.26% (*n* = 184/3497; CI 95%:4–6), 5.31% in 2022–2023 and 5.21% in 2023–2024, respectively. Positive samples were detected during all months of the hunting season but with a higher percentage at the beginning of the hunting season (September–October) than at the end (January) (Figure 5).

**Figure 6:**
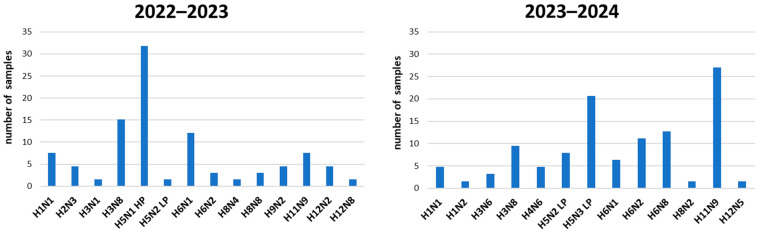
Distribution of AIV subtypes detected in the hunting seasons 2022–2023 and 2023–2024.

**Figure 7:**
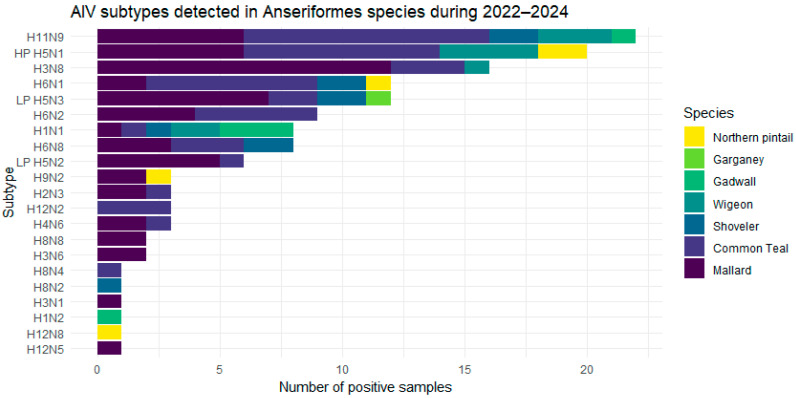
Distribution of AIV subtypes detected in wild duck species in the period 2022–2024.

Complete sequencing was obtained for 136 samples and showed the presence of 21 different subtypes from H1N1 to H12N5 (Figure 6); noteworthy, subtypes belonging to H7 were never detected during the study period. Different epidemiological scenarios were observed in the different years. Interestingly, the H5N1 HPAIV subtype, in particular of clade 2.3.4.4b, was the most frequent (31.8%) in 2022–2023 but was completely absent in winter 2023–2024 despite being widespread throughout Europe. In contrast, the H5 LPAIV strains, which were characterised by a low positivity rate (1.5%) in the hunting season 2022–2023, were the most frequently identified in 2023–2024 (28.6%). As H5 LPAI, H5N2 and H5N3 were detected. Almost all of these subtypes were detected in September 2023, and the subtype H5N3 resulted the prevalent. Among the other subtypes, H3N8, H6N1, H6N3, H6N8 and H11N9 were the most frequently detected (Figure 6). Figure 7 shows all subtypes detected, categorized by wild duck species sampled during the study period. The species with the highest variety of subtypes were teal and mallard, which were also the most frequently sampled species.

### 3.2. Surveillance in Parks

A total of 2031 faeces samples were collected from the ground, 1200 in 2022, 499 in 2023 and 332 in the first seven months of 2024, respectively. The samples were analysed in pools of up to five samples for a total of 487 pools analysed (281 pools in 2022, 124 in 2023 and 82 in 2024). All samples were negative for AIVs.

### 3.3. Passive Surveillance in Wild Bird Carcasses

**Figure 8:**
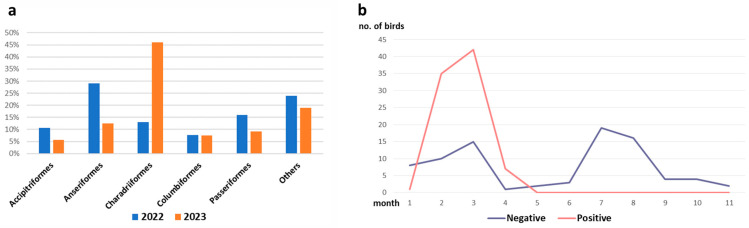
Passive surveillance in wild birds. (**a**) Percentage of collected birds divided by order; (**b**) number of gulls tested in 2023 divided by negative and positive for influenza type A.

The number of wild birds found and delivered to the IZSLER amounted to 481, of which 131 were in 2022 and 350 in 2023. The birds belonged to different orders; the most representative were *Charadriiformes* (37%), *Anseriformes* (17%), *Passeriformes* (11%), *Accipitriformes* (7%) and *Columbiformes* (7%). The remaining 21% consisted of 10 other orders characterised by low numbers of birds (Figure 8a). The molecular examinations revealed four H5N1 HPAIV clade 2.3.4.4b positive birds: two buzzards positive in 2022 (2/14, *p* = 14%), one grey heron (1/18, *p* = 5.5%) and one kestrel in 2023 (1/13, *p* = 7.6%). Concerning the order *Charadriiformes*, the gulls sampled were all negative for AIVs in 2022 (17 specimens), while 85 out of 167 individuals were positive in early 2023, showing a total prevalence of 51%. It should be noted that these positivities were all observed between February and April when the percentage of positives rose to 76% (Figure 8b).

Considering all the individual samples collected during this study (*n =* 3978), and excluding the faeces sampled within parks because it is impossible to determine the real numerical consistency of the animals involved, the relative percentages of positivity for each subtype detected are summarized in Table 1.

### 3.4. Phylogenetic Analyses

Phylogenetic analyses of the eight segments of 32 HPAI H5N1 viruses, for which the complete genome was obtained, indicated that all viruses collected from common gulls in 2023 (*n* = 24) belonged to H5N1 HPAIV clade 2.3.4.4b virus of the EA-2022-BB genotype [31], widely present throughout Europe in 2023 and characterised by high pathogenicity for colony-breeding seabird species (figures of phylogenetic trees are available in Supplementary Materials, from Figures S1–S8; the GISAID codes and genotype for H5N1 HPAIs are reported in Supplementary Table S1). The same genotype affected also three raptors (two peregrine falcons and one common kestrel) during the same time period. Differently, the H5N1 viruses (*n* = 4) collected from different wild duck species between October and December 2022 belonged to genotype EA-2021-AB. The topology of the phylogenetic trees clearly indicates that these viruses form two different clusters, suggesting at least two separate introductions of the EA-2021-AB genotype in the region. Sequences of LPAI H5N2 and H5N3 viruses cluster separately in the phylogenetic trees and are not related to the HPAI H5N1 viruses, suggesting the occurrence of no reassortment events between the co-circulating LPAI and HPAI strains in the monitored period. Blast analysis results for H and NA of all subtypes, excluding H5, are reported in Supplementary Tables S2 and S3.

## 4. Discussion

Using different sampling methods and biological matrices, this study allowed us to better evaluate the epidemiological situation regarding the circulation of avian influenza viruses in the Lombardy region. The data obtained from the cloacal swabs of hunted anatids show a rather complex picture, with the circulation of many different subtypes, probably resulting from different viral introductions and multiple reassortment events among wild waterfowl. The situation described is not unusual; in Europe, several studies conducted on wild birds highlighted the detection of different subtypes of AIV, especially in Northern Europe [32]. The most common subtypes detected were H4N6, H1N1, H2N3, H5N2, H6N2 and H11N9 [33,34]. Similarly, our study found that subtypes H11N9, H3N8, H6N1, H6N2, H1N1, H6N8 and H5N1 were the most frequently detected. Regarding the type of species infected, the pattern is also similar to previous research: mallard and Eurasian teal represent the species with the highest detected variability of subtypes [8,35,36]. However, the two species are also the most sampled, both because they are the most hunted species and due to their established role as the main reservoirs of the disease.

The variety of subtypes identified demonstrates, as is already known, how plastic this virus is and how it is capable of undergoing numerous mutations and reassortment events. Diversifying the ‘genetic heritage’ is indeed a successful survival strategy for all organisms. Having found so many different viruses in very common species shows how well the virus can adapt to its hosts and environment.

No viruses belonging to the H7 subtype have been observed among all subtypes identified. Viral strains belonging to this subtype are capable of infecting a wide range of domestic and wild avian susceptible species, circulating mainly sub-clinically as H7 LPAIV, but holding the capacity to evolve into HPAIV resulting in severe systemic disease and high mortality rates [37]. Several studies have confirmed a low frequency of identification of LPAIV H7 subtypes in wild birds, especially in long-term studies [33,38,39]. However, the short epidemic waves of H7 that have been detected over the years mainly occurred at the turn of winter and summer [39], i.e., a time window slightly different from the one considered in this study. Therefore, sampling only during the hunting season could lead to an underestimation of its presence for this subtype.

Linking sampling to the hunting season causes a bias in the sample collection, which is then carried out in a limited autumn/winter window. Nevertheless, several studies have demonstrated that the prevalence of AIVs, particularly in Mediterranean countries, is markedly reduced during the spring and early summer months in comparison to the autumn and winter seasons [32,34]. It is, therefore, possible to assume that this sampling window is likely to be representative and gives a rather reliable general picture of the circulation of the different subtypes in the wild duck population. This seasonal spread of the virus could also be linked to migration flows. In particular, the autumnal migration of waterfowl, including hatch-year birds, which are more susceptible to infection, can increase the transmission of the HPAIV H5 virus [9,40].

With regard to H5 strains, the massive spread of the H5N1 subtype occurred in the last years in colony-breeding seabirds [24,41], usually characterised by relatively silent periods in warm seasons, has resulted in high peaks of infection even during spring and summer periods. The results presented here on the H5 subtypes detected in hunted ducks showed a different epidemiological situation between the two years. Note that they were detected during all the hunting seasons but with higher frequency in the first months (September and October). In the season 2022–2023, the most isolated subtype was H5N1 HPAIV, reflecting the situation across Europe. The same strain was never identified in Lombardy in autumn and winter 2023. The cases reported in 2022–2023 in the restricted regional area could be seen as the aftermath of the great epidemic wave that began around 2020, which affected many wild and domestic bird species and several mammal species worldwide [18,42]. Furthermore, the presence of clade 2.3.4.4b in the sampled avian species places particular emphasis on the possible risk of these birds spreading viruses to humans, especially for hunters, and their domestic animals (dogs, cats, cattle and captive birds used as decoys). Indeed, since 2022, sporadic human infections with H5 HPAI have been reported, recently associated with clade 2.3.4.4b in several countries in Europe, South and North America and Asia. Almost all reported cases were due to recent exposure to sick or dead poultry even if some were considered asymptomatic. It is possible that these cases only represented contamination of the respiratory tract without productive infections [43]. In addition, 14 human cases were associated with exposure to infected birds, poultry and dairy cows in the USA; nine were confirmed as H5N1 [37]. The total absence of this strain during the winter season of 2023–2024 in Lombardy is noteworthy despite its documented presence and circulation in Europe during the same period. However, these data are in line with the progressive decrease in H5 HPAIV observed in wild birds in European countries since the second half of 2023 [13]. During this period, the most represented strains were H5 LPAIVs. However, the pattern found is somewhat anomalous. Considering the global situation, it can be seen that, actually, in the same study period, there was an identification of H5 LPAIV strains only towards the end of 2023. Moreover, reports in Europe are almost absent, and most of the H5 LPAIV outbreaks in wild animals have occurred in eastern Asia (data from EMPRES-i FAO report dates from 1 January 2022 to October 2024). If the surveillance carried out on hunted animals confirms the central role of Anatidae in the maintenance and spread of the AI viruses, the data obtained through passive surveillance on animals found dead confirm a significant role for birds belonging to the order *Charadriiformes* in spreading a specific genotype of H5 HPAIVs. Indeed, 85 gulls out of 167 resulted positive since early 2023 to the strain H5N1, clade 2.3.4.4b, genotype EA-2022-BB [3,44]. Phylogenetic studies have shown that the genotype EA-2022-BB resulted from a reassortment between an H5N1 HPAI virus and a low pathogenicity subtype (LPAI) particularly adapted to gulls (H13) [3,4] and, therefore, was particularly widespread among birds of the *Laridae* family. This genotype emerged in Europe in 2022 and was detected in Italy in 2023, causing some outbreaks in poultry farms in Northern Italy [3,44]. With regard to black-headed gulls (*Chroicocephalus ridibundus*) in particular, there has been a veritable die-off of these birds due to the H5N1 strain since early 2023; it is possible that these mass mortality events, recorded in Italy for the first time, were caused by the entry of infected migratory birds from northern Europe during December 2022 [45,46]. The disease spread very quickly in Lombardy, and the mortality rate was almost all concentrated in the first months of the year, immediately after the virus had passed through the gull population. Looking at the months from February to April 2023, mortality and AIV positivity rates were close to 76%, followed by a drastic reduction in cases in the following months [47].

The sampling derived from passive surveillance and the collection of duck faeces within the parks helped to extend monitoring throughout the year, partially complementing the sampling linked to hunting activities that are time-restricted. However, at least for the present study, data provided by faecal sampling within the parks added no further information on virus circulation, even though the selected parks were characterised by suitable environments for reservoir species. Previous monitoring surveys conducted in 2021, by applying the same procedures used in the present study, had allowed the detection of influenza viruses (unpublished data) in support of the validity of the biological matrix chosen for analysis, albeit in a lower percentage (3.8%: 3 out of 77 positive pool untyped) than in the sampled hunted ducks. It may be assumed that to some extent, the environmental conditions were not always optimal for the survival of the virus, causing its degradation, or that the faecal matrix, collected and analysed in a pool, underwent a sort of dilution of the virus, which was, therefore, present in quantities below the detection threshold.

Passive surveillance of dead animals highlighted the high mortality in gulls and identified other AIV-positive birds. These included buzzards, herons and kestrels. The positivity found in birds of prey should be investigated further; it is presumable that, being predators and highly susceptible to infection, they obtained the infection from dead or sick prey, thus acting as sentinels of the diseases occurring in other bird species [48,49]. However, the increase in the number of species involved in the spread of the disease, observed in recent years, should draw attention to any changes that may be taking place in the epidemiological cycle of HPAI viruses.

## 5. Conclusions

Active surveillance of wild birds, particularly those that can silently maintain HPAI viruses in the wild, such as wild ducks, is strategic to enhance knowledge of HPAI and LPAI viruses circulating in a specific area.

In areas with a high density of poultry, preparedness and prevention strategies should consistently be implemented to mitigate the risk of introduction in commercial farms and secondary spread, as well as measures to safeguard public health. At the same, the early identification of HPAI viruses circulating in the wild through the application of specific surveillance plans focused on wild birds allows for strengthening such prevention measures and elevating the level of attention to detect even more promptly possible outbreaks in farmed poultry and in-contact animals and people. One supporting data point for early detection is the fact that the first detection of H5N1HPAIV in domestic poultry in Lombardy in 2021 was reported after detection in wild animals and that genomic sequencing confirmed that the first case resulted from an introduction from the wild bird [50,51].

The timely generation and sharing of genome sequence data for avian influenza viruses, not only those of high pathogenicity, is of paramount importance. This enables an early identification of viruses with mutations associated with increased zoonotic potential, resistance to antiviral drugs or unusual antigenic properties. It also provides a comprehensive view of the subtypes circulating in a given territory and the genetic features of the circulating genotypes.

## Figures and Tables

**Table 1:** Percentages of positivity are reported for each subtype detected. Samples from parks were excluded.

Subtype	Number of Birds	% Positivity
H1N1	8	0.20%
H1N2	1	0.03%
H2N3	3	0.08%
H3N1	1	0.03%
H3N6	2	0.05%
H3N8	16	0.40%
H4N6	3	0.08%
H5N3	1	0.03%
H6N1	12	0.30%
H6N2	9	0.23%
H6N8	8	0.20%
H8N2	1	0.03%
H8N4	1	0.03%
H8N8	2	0.05%
H9N2	3	0.08%
H11N9	22	0.55%
H12N2	3	0.08%
H12N5	1	0.03%
H12N8	1	0.03%
HP H5N1	109	2.74%
LP H5N2	6	0.15%
LP H5N3	11	0.28%

## Data Availability

The original contributions presented in the study are included in the article/Supplementary Materials, further inquiries can be directed to the corresponding author.

## Supplementary Materials

The following supporting information can be downloaded at: https://www.mdpi.com/article/10.3390/v16111668/s1, Figure S1: Phylogenetic tree of H5 AIVs based on PB2 gene; Figure S2: Phylogenetic tree of H5 AIVs based on PB1 gene; Figure S3: Phylogenetic tree of H5 AIVs based on PA gene; Figure S4: Phylogenetic tree of H5 AIVs based on HA gene; Figure S5: Phylogenetic tree of H5 AIVs based on NP gene; Figure S6: Phylogenetic tree of H5 AIVs based on NA gene; Figure S7: Phylogenetic tree of H5 AIVs based on M gene; Figure S8: Phylogenetic tree of H5 AIVs based on NS gene; Table S1: Gisaid accession numbers and genetic characteristics for the H5 AIV samples; Table S2: Blast analysis of HA genes for all non-H5 samples; Table S3: Blast analysis of NA genes for all non-H5 samples.
