# Supplementary material for: Surveillance for Avian Influenza in Wild Birds in the Lombardy Region (Italy) in the Period 2022–2024
Source: Viruses. 2024 Oct 24;16(11):1668. doi: 10.3390/v16111668 (PMC11598995; doi:10.3390/v16111668)
Supplement: Supplementary file 1 [file viruses-16-01668-s001.zip › viruses-3204682-supplementary.pdf]

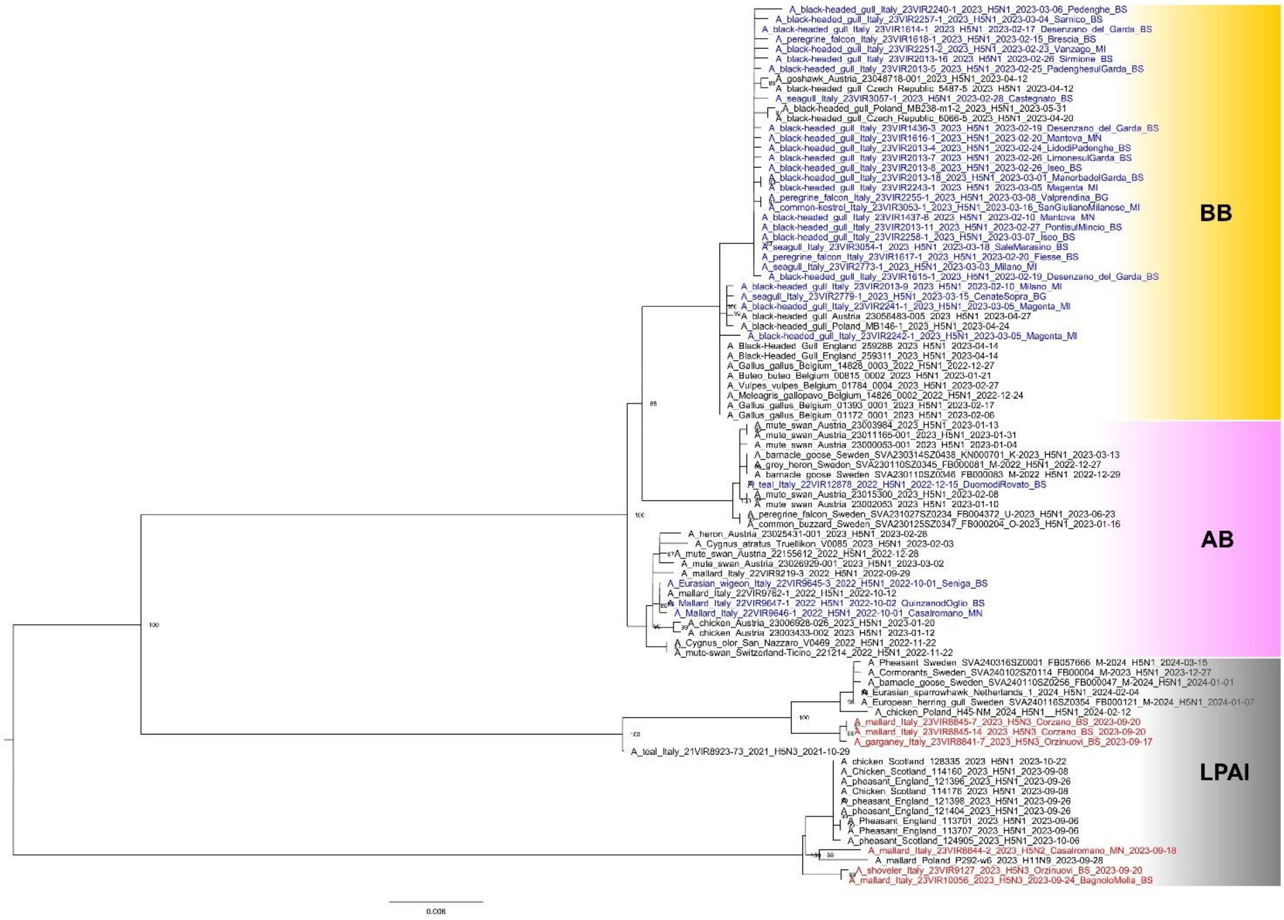

Figure S1. Phylogenetic tree of H5 AIVs based on PB2 gene.

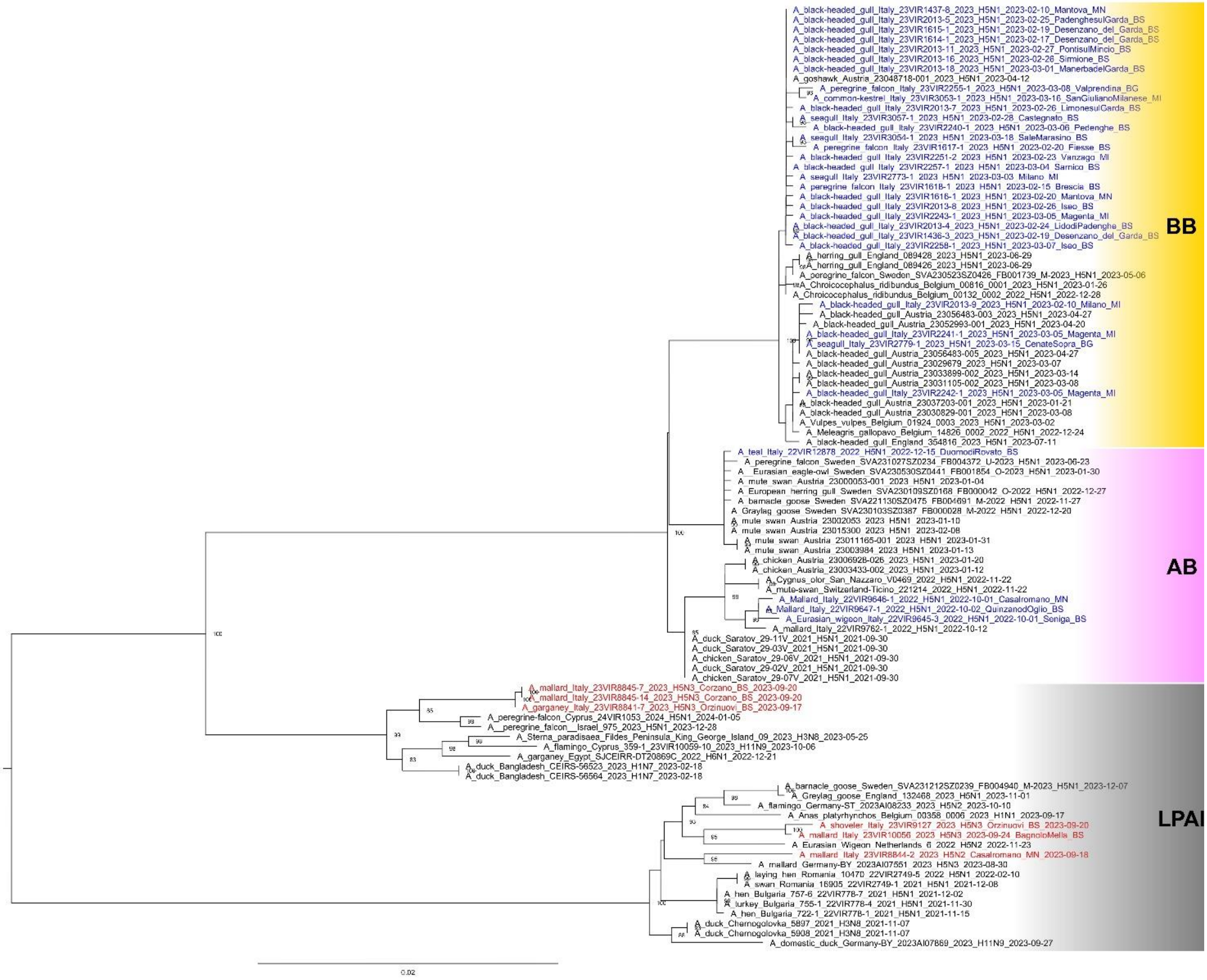

Figure S2. Phylogenetic tree of H5 AIVs based on PB1 gene.

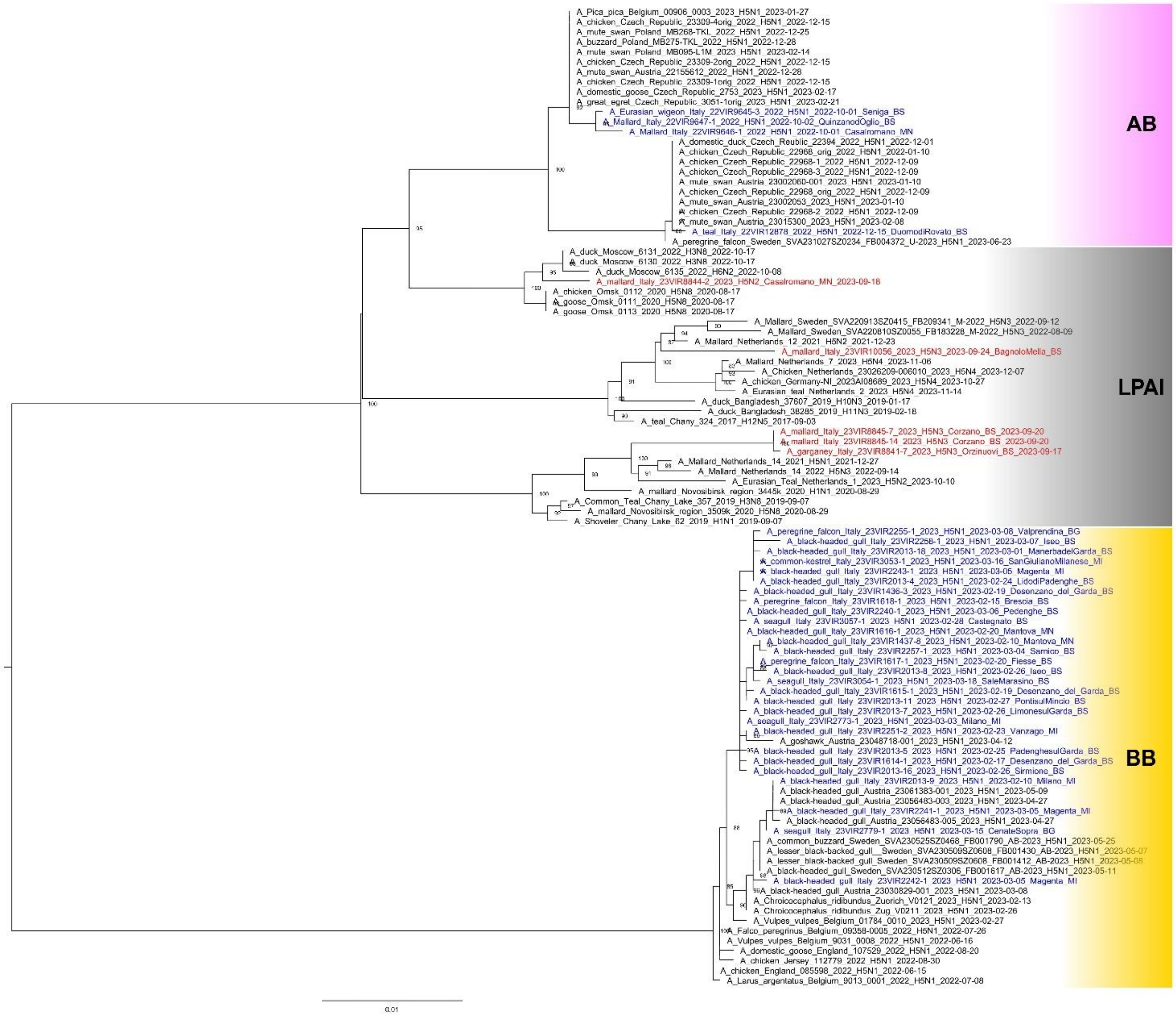

Figure S3. Phylogenetic tree of H5 AIVs based on PA gene.

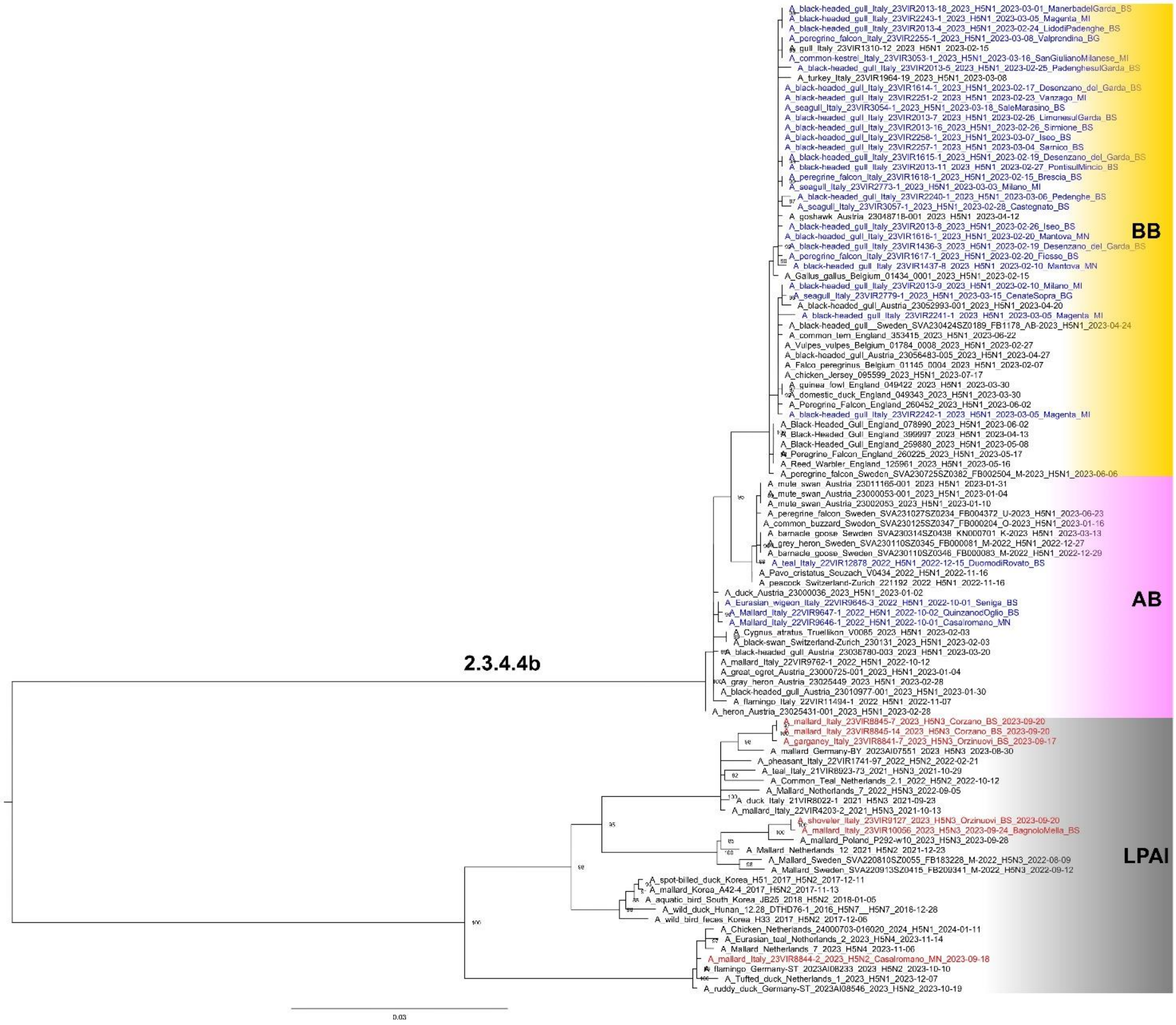

Figure S4. Phylogenetic tree of H5 AIVs based on HA gene.

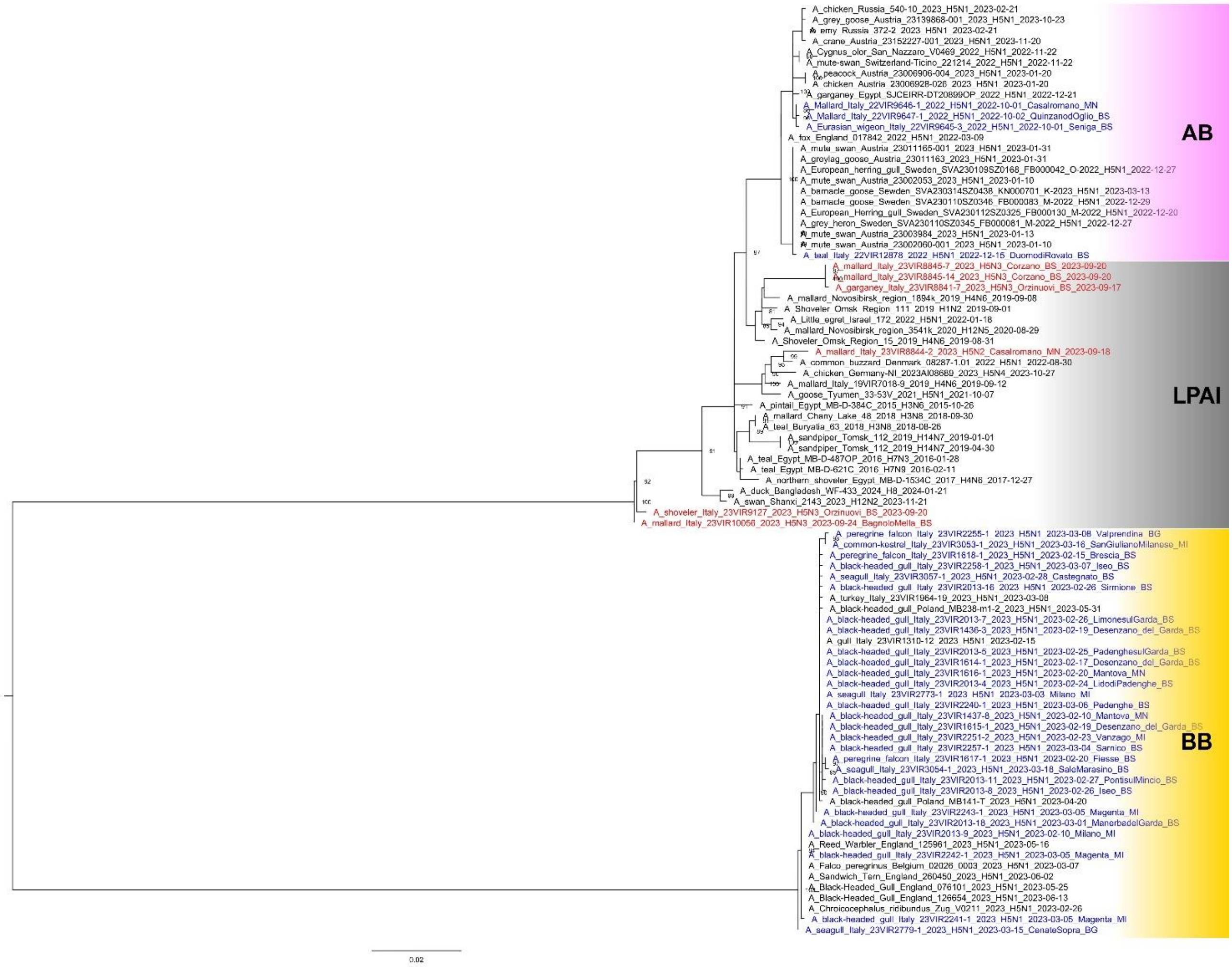

Figure S5. Phylogenetic tree of H5 AIVs based on NP gene.

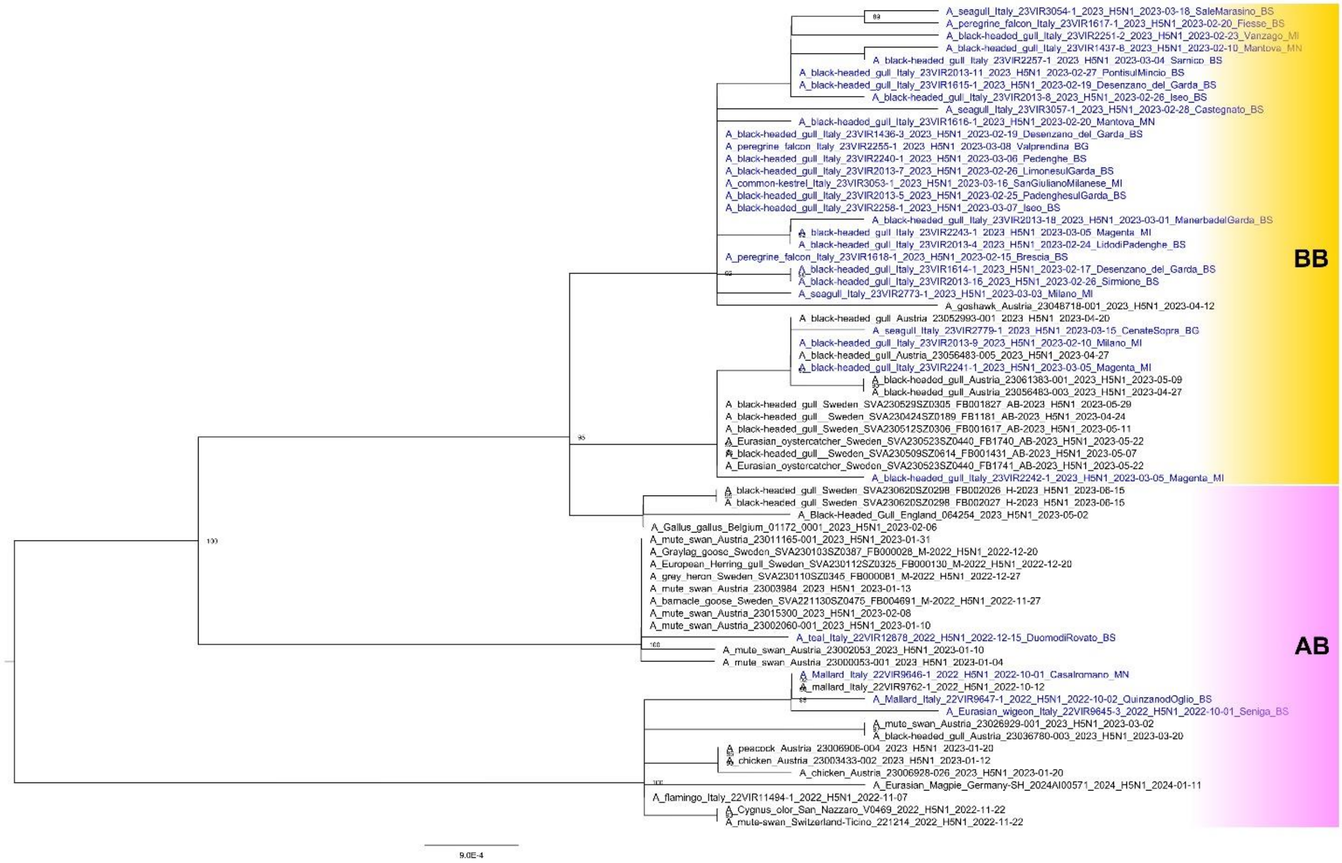

Figure S6. Phylogenetic tree of H5 AIVs based on NA gene.

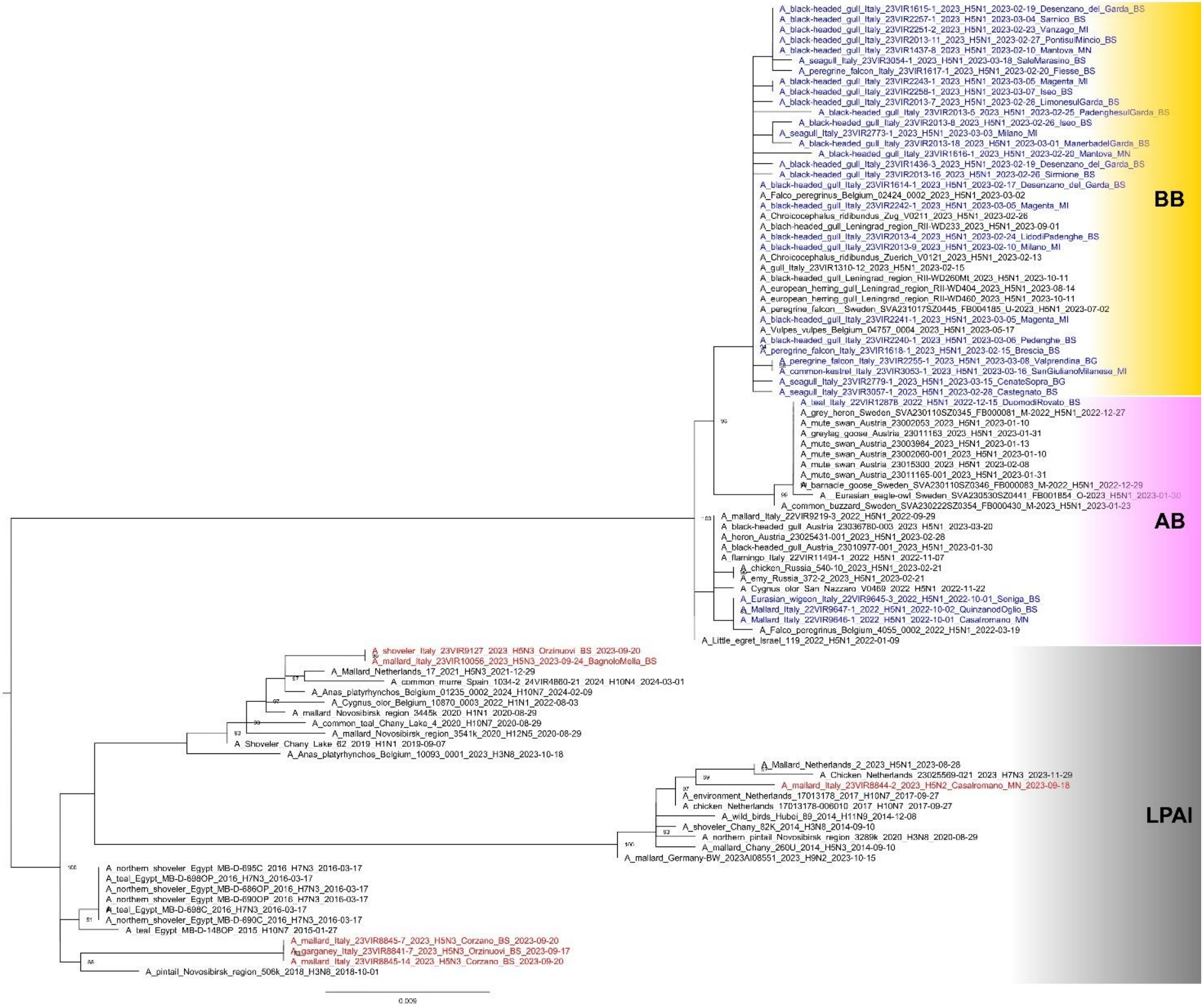

Figure S7. Phylogenetic tree of H5 AIVs based on M gene.

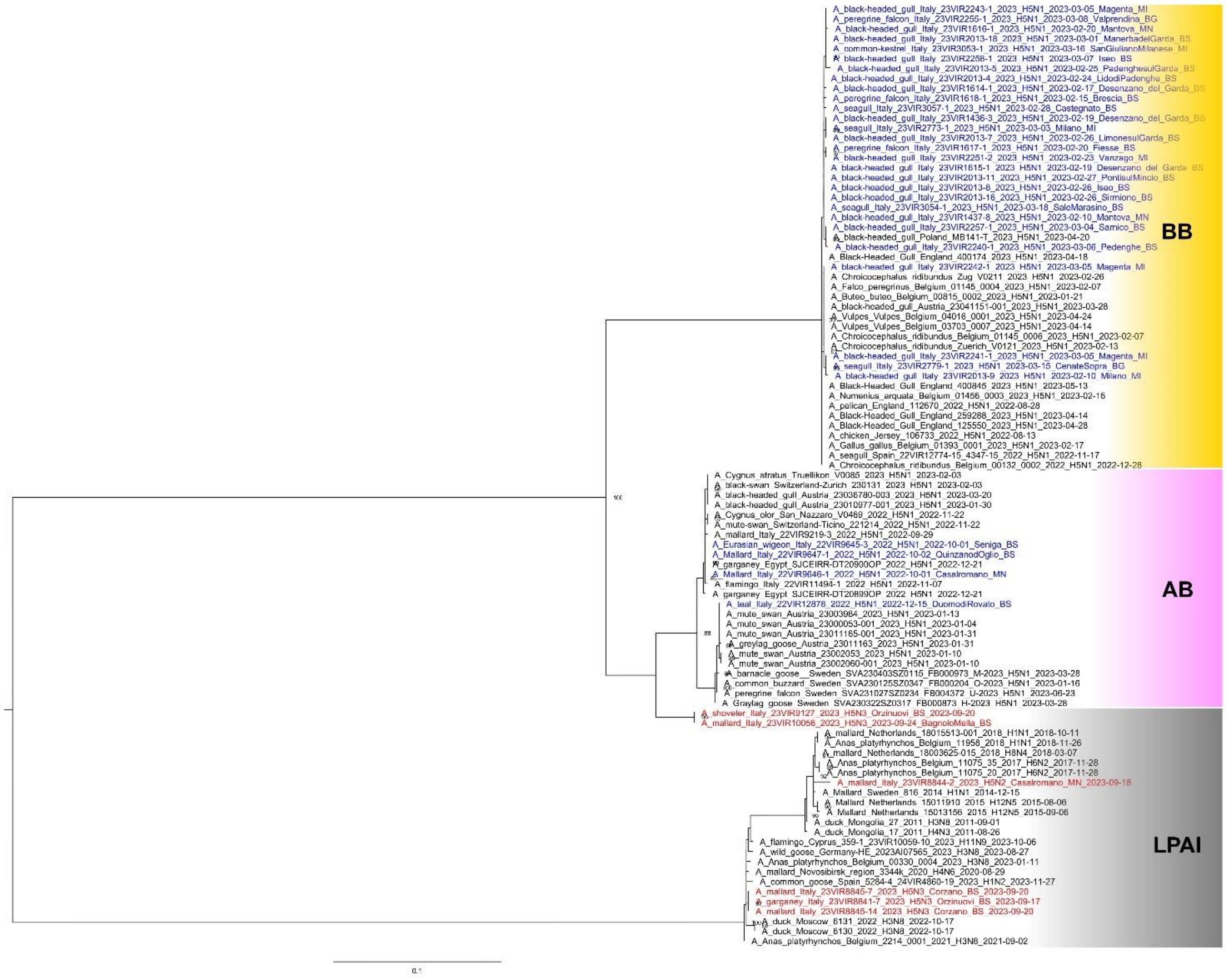

Figure S8. Phylogenetic tree of H5 AIVs based on NS gene.

Table S1. Gisaid accession numbers and genetic characteristics for the H5 AIV samples.

Strain	Gisaid_ID	Genotype	PB2	PB1	PA	HA	NP	NA	MP	NS
>A/Eurasian_wigeon/Italy/22VIR9645-3/2022_H5N1_2022-10-01_Seniga_BS	19462778	AB	31	1	3	20	38	1	20	1
>A/Mallard/Italy/22VIR9646-1/2022_H5N1_2022-10-01_Casalromano_MN	19462779	AB	31	1	3	20	38	1	20	1
>A/Mallard/Italy/22VIR9647-1/2022_H5N1_2022-10-02_QuinzanodOglio_BS	19462780	AB	31	1	3	20	38	1	20	1
>A/teal/Italy/22VIR12878/2022_H5N1_2022-12-15_DuomodiRovato_BS	19462781	AB	31	1	3	20	38	1	20	1
>A/black-headed_gull/Italy/23VIR1437-8/2023_H5N1_2023-02-10_Mantova_MN	19462782	BB	31	1	43	20	43	1	20	43
>A/black-headed_gull/Italy/23VIR2013-9/2023_H5N1_2023-02-10_Milano_MI	19462807	BB	31	1	43	20	43	1	20	43
>A/peregrine_falcon/Italy/23VIR1618-1/2023_H5N1_2023-02-15_Brescia_BS	19462795	BB	31	1	43	20	43	1	20	43
>A/black-headed_gull/Italy/23VIR1614-1/2023_H5N1_2023-02-17_Desenzano_del_Garda_BS	19462784	BB	31	1	43	20	43	1	20	43
>A/black-headed_gull/Italy/23VIR1436-3/2023_H5N1_2023-02-19_Desenzano_del_Garda_BS	19462783	BB	31	1	43	20	43	1	20	43
>A/black-headed_gull/Italy/23VIR1615-1/2023_H5N1_2023-02-19_Desenzano_del_Garda_BS	19462792	BB	31	1	43	20	43	1	20	43
>A/black-headed_gull/Italy/23VIR1616-1/2023_H5N1_2023-02-20_Mantova_MN	19462785	BB	31	1	43	20	43	1	20	43
>A/peregrine_falcon/Italy/23VIR1617-1/2023_H5N1_2023-02-20_Fiesse_BS	19462794	BB	31	1	43	20	43	1	20	43
>A/black-headed_gull/Italy/23VIR2251-2/2023_H5N1_2023-02-23_Vanzago_MI	19462788	BB	31	1	43	20	43	1	20	43
>A/black-headed_gull/Italy/23VIR2013-4/2023_H5N1_2023-02-24_LidodiPadenghe_BS	19462798	BB	31	1	43	20	43	1	20	43

>A/black-headed_gull/Italy/23VIR2013-5/2023_H5N1_2023-02-25_PadenghesulGarda_BS	19462805	BB	31	1	43	20	43	1	20	43
>A/black-headed_gull/Italy/23VIR2013-7/2023_H5N1_2023-02-26_LimonesulGarda_BS	19462786	BB	31	1	43	20	43	1	20	43
>A/black-headed_gull/Italy/23VIR2013-8/2023_H5N1_2023-02-26_Iseo_BS	19462803	BB	31	1	43	20	43	1	20	43
>A/black-headed_gull/Italy/23VIR2013-16/2023_H5N1_2023-02-26_Sirmione_BS	19462787	BB	31	1	43	20	43	1	20	43
>A/black-headed_gull/Italy/23VIR2013-11/2023_H5N1_2023-02-27_PontisulMincio_BS	19462793	BB	31	1	43	20	43	1	20	43
>A/seagull/Italy/23VIR3057-1/2023_H5N1_2023-02-28_Castegnato_BS	19462806	BB	31	1	43	20	43	1	20	43
>A/black-headed_gull/Italy/23VIR2013-18/2023_H5N1_2023-03-01_ManerbadelGarda_BS	19462799	BB	31	1	43	20	43	1	20	43
>A/seagull/Italy/23VIR2773-1/2023_H5N1_2023-03-03_Milano_MI	19462796	BB	31	1	43	20	43	1	20	43
>A/black-headed_gull/Italy/23VIR2257-1/2023_H5N1_2023-03-04_Sarnico_BS	19462789	BB	31	1	43	20	43	1	20	43
>A/black-headed_gull/Italy/23VIR2241-1/2023_H5N1_2023-03-05_Magenta_MI	19462810	BB	31	1	43	20	43	1	20	43
>A/black-headed_gull/Italy/23VIR2242-1/2023_H5N1_2023-03-05_Magenta_MI	19462809	BB	31	1	43	20	43	1	20	43
>A/black-headed_gull/Italy/23VIR2243-1/2023_H5N1_2023-03-05_Magenta_MI	19462800	BB	31	1	43	20	43	1	20	43
>A/black-headed_gull/Italy/23VIR2240-1/2023_H5N1_2023-03-06_Pedenghe_BS	19462804	BB	31	1	43	20	43	1	20	43
>A/black-headed_gull/Italy/23VIR2258-1/2023_H5N1_2023-03-07_Iseo_BS	19462790	BB	31	1	43	20	43	1	20	43
>A/peregrine_falcon/Italy/23VIR2255-1/2023_H5N1_2023-03-08_Valprendina_BG	19462801	BB	31	1	43	20	43	1	20	43
>A/seagull/Italy/23VIR2779-1/2023_H5N1_2023-03-15_CenateSopra_BG	19462808	BB	31	1	43	20	43	1	20	43
>A/common-kestrel/Italy/23VIR3053-1/2023_H5N1_2023-03-16_SanGiulianoMilanese_MI	19462802	BB	31	1	43	20	43	1	20	43
>A/seagull/Italy/23VIR3054-1/2023_H5N1_2023-03-18_SaleMarasino_BS	19462791	BB	31	1	43	20	43	1	20	43
>A/mallard/Italy/23VIR8845-7/2023_H5N3_Corzano_BS_2023-09-20	18473722	LPAI								
>A/shoveler/Italy/23VIR9127/2023_H5N3_Orzinuovi_BS_2023-09-20	18473723	LPAI								
>A/mallard/Italy/23VIR8845-14/2023_H5N3_Corzano_BS_2023-09-20	18473721	LPAI								
>A/garganey/Italy/23VIR8841-7/2023_H5N3_Orzinuovi_BS_2023-09-17	18612187	LPAI								
>A/mallard/Italy/23VIR8844-2/2023_H5N2_Casalromano_MN_2023-09-18	18612267	LPAI								
>A/mallard/Italy/23VIR10056/2023_H5N3_2023-09-24_BagnoloMella_BS	19463386	LPAI								

Table S2. Blast analysis of HA genes for all non-H5 samples.

Sample	HA	% identity	Closest sequence	Accession no.
A/Mallard/Italy/23013/2022_HA_H1N1	H1	98.17	A/northern pintail duck/Egypt/MB-D-2211C/2019(H1N1)	OQ797596
A/Shoveler/Italy/23061/2022_HA_H1N1	H1	98.00	A/northern pintail duck/Egypt/MB-D-2211C/2019(H1N1)	OQ797596
A/Wigeon/Italy/367477_2/2022_HA_H1N1	H1	97.74	A/northern pintail duck/Egypt/MB-D-2211C/2019(H1N1)	OQ797596
A/Wigeon/Italy/367477_3/2022_HA_H1N1	H1	97.74	A/northern pintail duck/Egypt/MB-D-2211C/2019(H1N1)	OQ797596
A/Common_teal/Italy/326040_1/2022_HA_H1N1	H1	98.29	A/northern pintail duck/Egypt/MB-D-2211C/2019(H1N1)	OQ797596
A/Gadwall/Italy/386681_1/2023_HA_H1N1	H1	98.29	A/northern pintail duck/Egypt/MB-D-2211C/2019(H1N1)	OQ797596
A/Gadwall/Italy/386681_2/2023_HA_H1N1	H1	98.29	A/northern pintail duck/Egypt/MB-D-2211C/2019(H1N1)	OQ797596
A/Gadwall/Italy/386681_3/2023_HA_H1N1	H1	98.29	A/northern pintail duck/Egypt/MB-D-2211C/2019(H1N1)	OQ797596
A/Gadwall/Italy/394037_4/2023_HA_H1N2	H1	98.12	A/northern pintail duck/Egypt/MB-D-2211C/2019(H1N1)	OQ797596
A/Mallard/Italy/355612_1/2022_HA_H2N3	H2	97.03	A/common teal/Egypt/MB-P-1352C/2017(H2N3)	OQ797640.1
A/Mallard/Italy/355612_2/2022_HA_H2N3	H2	97.15	A/common teal/Egypt/MB-P-1352C/2017(H2N3)	OQ797640.1
A/Common_teal/Italy/330815_3/2022_HA_H2N3	H2	97.15	A/common teal/Egypt/MB-P-1352C/2017(H2N3)	OQ797640.1
A/Mallard/Italy/338541_4/2022_HA_H3N1	H3	98.88	A/duck/Moscow/5881/2021(H3N2)	OP133624.1
A/Mallard/Italy/321045_3/2022_HA_H3N8	H3	99.42	A/duck/Moscow/6134/2022(H3N8)	PP897264.1

A/Mallard/Italy/321045_4/2022_HA_H3N8	H3	99.47	A/duck/Moscow/6134/2022(H3N8)	PP897264.1
A/Mallard/Italy/321045_6/2022_HA_H3N8	H3	99.41	A/duck/Moscow/6134/2022(H3N8)	PP897264.1
A/Mallard/Italy/321045_7/2022_HA_H3N8	H3	99.47	A/duck/Moscow/6134/2022(H3N8)	PP897264.1
A/Mallard/Italy/321045_8/2022_HA_H3N8	H3	99.41	A/duck/Moscow/6134/2022(H3N8)	PP897264.1
A/Common_teal/Italy/321099_1/2022_HA_H3N8	H3	99.47	A/duck/Moscow/6134/2022(H3N8)	PP897264.1
A/Common_teal/Italy/326485/2022_HA_H3N8	H3	99.41	A/duck/Moscow/6134/2022(H3N8)	PP897264.1
A/Mallard/Italy/321045_1/2022_HA_H3N8	H3	99.47	A/duck/Moscow/6134/2022(H3N8)	PP897264.1
A/Mallard/Italy/321045_2/2022_HA_H3N8	H3	99.47	A/duck/Moscow/6134/2022(H3N8)	PP897264.1
A/Mallard/Italy/321045_5/2022_HA_H3N8	H3	99.47	A/duck/Moscow/6134/2022(H3N8)	PP897264.1
A/Mallard/Italy/302598_2/2023_HA_H3N6	H3	98.00	A/duck/Mongolia/876/2019(H3N8)	MT020270.1
A/Mallard/Italy/302598_4/2023_HA_H3N6	H3	98.00	A/duck/Mongolia/876/2019(H3N8)	MT020270.1
A/Mallard/Italy/299740_5/2023_HA_H3N8	H3	98.12	A/duck/Mongolia/876/2019(H3N8)	MT020270.1
A/Mallard/Italy/304157_1/2023_HA_H3N8	H3	98.06	A/duck/Mongolia/876/2019(H3N8)	MT020270.1
A/Mallard/Italy/321660_1/2023_HA_H3N8	H3	98.12	A/duck/Assam/DUOR1512100004/2015(H3N8)	MT272421.1
A/Mallard/Italy/321667_2/2023_HA_H3N8	H3	98.12	A/duck/Assam/DUOR1512100004/2015(H3N8)	MT272421.1
A/Common_teal/Italy/336752_4/2023_HA_H3N8	H3	97.88	A/duck/Mongolia/876/2019(H3N8)	MT020270.1
A/Wigeon/Italy/336772_1/2023_HA_H3N8	H3	97.88	A/duck/Mongolia/876/2019(H3N8)	MT020270.1
A/Wigeon/Italy/336772_2/2023_HA_H3N8	H3	97.88	A/duck/Mongolia/876/2019(H3N8)	MT020270.1
A/Common_teal/Italy/386684_3/2023_HA_H4N6	H4	97.93	A/duck/Bangladesh/50268/2021(H4N6)	OP023519.1
A/Mallard/Italy/321660_2/2023_HA_H4N6	H4	98.17	A/duck/Bangladesh/50268/2021(H4N6)	OP023519.1
A/Eurasian_wigeon/Italy/22VIR9645_3/2022_HA_H5N1_Seniga	H5	99.76	A/Cygnus atratus/Truellikon/V0085_HA/2023(H5N1)	PQ098765.1
A/Mallard/Italy/348683_1/2022_HA_H5N2	H5	96.22	A/aquatic bird/South Korea/JB25/2018(H5N2)	OP905574.1
A/Mallard/Italy/22VIR9646_1/2022_HA_H5N1_Casalromano	H5	99.71	A/Cygnus atratus/Truellikon/V0085_HA/2023(H5N1)	PQ098765.1
A/Mallard/Italy/22VIR9909_1/2022_HA_H5N1_Quinzano	H5	99.71	A/Cygnus atratus/Truellikon/V0085_HA/2023(H5N1)	PQ098765.1
A/Teal/Italy/22VIR11684_2/2022_HA_H5N1_DuomodiRovato	H5	99.94	A/Pavo cristatus/Seuzach/V0434_HA/2022(H5N1)	PQ098776.1
A/Teal/Italy/22VIR12727_1/2022_HA_H5N1_DuomodiRovato	H5	99.82	A/Pavo cristatus/Seuzach/V0434_HA/2022(H5N1)	PQ098776.1
A/Teal/Italy/22VIR12878/2022_HA_H5N1_DuomodiRovato	H5	99.82	A/Pavo cristatus/Seuzach/V0434_HA/2022(H5N1)	PQ098776.1
A/Shoveler/Italy/306797_2/2023_HA_H5N3	H5	96.51	A/gadwall/Ningxia/YG61/2017(H5N6)	MH283021.1
A/Teal/Italy/399256/2022_HA_H6N2	H6	99.29	A/garganey/Egypt/DT20869C/2022(H6N1)	OR786180.1
A/Teal/401707/2022_HA_H6N2	H6	99.41	A/duck/Moscow/6135/2022(H6N2)	PP897280.1
A/Teal/Italy/413742/2022_HA_H6N1	H6	98.88	A/garganey/Egypt/DT20869C/2022(H6N1)	OR786180.1
A/Mallard/Italy/390956_2/2022_HA_H6N1	H6	99.23	A/duck/Moscow/6135/2022(H6N2)	PP897280.1
A/Mallard/Italy/390906_1/2022_HA_H6N1	H6	99.00	A/Pekin_duck/France/21114/2021(H6N1)	OP828914.1
A/Mallard/Italy/390906_3/2022_HA_H6N1	H6	98.94	A/garganey/Egypt/DT20869C/2022(H6N1)	OR786180.1
A/Northern_shoveler/Italy/390877_2/2022_HA_H6N1	H6	99.35	A/garganey/Egypt/DT20869C/2022(H6N1)	OR786180.1
A/Northern_shoveler/Italy/390877_3/2022_HA_H6N1	H6	99.35	A/garganey/Egypt/DT20869C/2022(H6N1)	OR786180.1
A/Common_teal/Italy/390919_8/2022_HA_H6N1	H6	99.00	A/Pekin_duck/France/21114/2021(H6N1)	OP828914.1
A/Common_teal/Italy/347389_6/2022_HA_H6N1	H6	98.65	A/duck/Moscow/6135/2022(H6N2)	PP897280.1
A/Mallard/Italy/407002_2/2023_HA_H6N8	H6	99.47	A/duck/Moscow/6135/2022(H6N2)	PP897280.1
A/Mallard/Italy/407002_3/2023_HA_H6N8	H6	99.47	A/duck/Moscow/6135/2022(H6N2)	PP897280.1
A/Common_teal/Italy/407012_4/2023_HA_H6N8	H6	99.47	A/duck/Moscow/6135/2022(H6N2)	PP897280.1
A/Common_teal/Italy/407012_5/2023_HA_H6N8	H6	99.57	A/duck/Moscow/6135/2022(H6N2)	PP897280.1

A/Mallard/Italy/299696_7/2023_HA_H6N2	H6	98.00	A/Arenaria interpres/Belgium/11446/2016(H6N8)	MT407137.1
A/Mallard/Italy/306879_4/2023_HA_H6N2	H6	97.94	A/Arenaria interpres/Belgium/11446/2016(H6N8)	MT407137.1
A/Mallard/Italy/315897_1/2023_HA_H6N2	H6	97.88	A/Arenaria interpres/Belgium/11446/2016(H6N8)	MT407137.1
A/Common_teal/Italy/320322_1/2023_HA_H6N2	H6	97.88	A/Arenaria interpres/Belgium/11446/2016(H6N8)	MT407137.1
A/Common_teal/Italy/342250_2/2023_HA_H6N2	H6	99.18	A/duck/Moscow/6135/2022(H6N2)	PP897280.1
A/Mallard/Italy/342238_2/2023_HA_H6N2	H6	97.82	A/Arenaria interpres/Belgium/11446/2016(H6N8)	MT407137.1
A/Northern_pintail/Italy/337645/2023_HA_H6N1	H6	99.18	A/duck/Moscow/6135/2022(H6N2)	PP897280.1
A/Common_teal/Italy/376566_2/2023_HA_H6N2	H6	97.82	A/Arenaria interpres/Belgium/11446/2016(H6N8)	MT407137.1
A/Mallard/Italy/379789_2/2023_HA_H6N8	H6	99.35	A/duck/Moscow/6135/2022(H6N2)	PP897280.1
A/Shoveler/Italy/392262_2/2023_HA_H6N8	H6	97.82	A/Arenaria interpres/Belgium/11446/2016(H6N8)	MT407137.1
A/Shoveler/Italy/392262_3/2023_HA_H6N8	H6	98.06	A/Arenaria interpres/Belgium/11446/2016(H6N8)	MT407137.1
A/Common_teal/Italy/407012_2/2023_HA_H6N8	H6	99.47	A/duck/Moscow/6135/2022(H6N2)	PP897280.1
A/Common_teal/Italy/407012_3/2023_HA_H6NX	H6	99.47	A/duck/Moscow/6135/2022(H6N2)	PP897280.1
A/Common_teal/Italy/362850_1/2022_HA_H8N4	H8	99.00	A/duck/Bangladesh/37509/2019(H8N4)	MT090424.1
A/Mallard/Italy/335123_7/2022_HA_H8N8	H8	98.23	A/duck/Bangladesh/37509/2019(H8N4)	MT090424.1
A/Mallard/Italy/335123_5/2022_HA_H8N8	H8	98.23	A/duck/Bangladesh/37509/2019(H8N4)	MT090424.1
A/Shoveler/Italy/394076/2023_HA_H8N2	H8	94.88	A/duck/Mongolia/565/2011(H8N4)	LC367418.1
A/teal/Italy/418637/2022_HA_H9N2	H9	97.80	A/duck/Bangladesh/44493/2020(H9N9)	MW749817.1
A/Mallard/Italy/328086_2/2022_HA_H9N2	H9	97.92	A/duck/Bangladesh/44493/2020(H9N9)	MW749817.1
A/Mallard/Italy/328091_2/2022_HA_H9N2	H9	97.92	A/duck/Bangladesh/44493/2020(H9N9)	MW749817.1
A/Northern_pintail/Italy/347406_5/2022_HA_H9N2	H9	97.80	A/duck/Bangladesh/44493/2020(H9N9)	MW749817.1
A/Teal/Italy/417152_1/2022_HA_H11N9	H11	98.64	A/duck/Moscow/5712U/2019(H11N6)	MW186787.1
A/Teal/Italy/422038_2/2022_HA_H11N9	H11	98.35	A/Anas platyrhynchos/South Korea/JB31-103/2019(H11N9)	MW116722.1
A/Mallard/Italy/432522_1/2022_HA_H11N9	H11	98.58	A/duck/Moscow/5712U/2019(H11N6)	MW186787.1
A/Mallard/Italy/4854_1/2023_HA_H11N9	H11	98.58	A/duck/Moscow/5712U/2019(H11N6)	MW186787.1
A/Mallard/Italy/359760/2022_HA_H11N9	H11	98.82	A/duck/Moscow/5712U/2019(H11N6)	MW186787.1
A/Gadwall/Italy/386681_4/2023_HA_H11N9	H11	99.29	A/duck/Moscow/6455/2023(H11N9)	PP897307.1
A/Mallard/Italy/386690_2/2023_HA_H11N9	H11	99.23	A/duck/Moscow/6455/2023(H11N9)	PP897307.1
A/Common_teal/Italy/395613_5/2023_HA_H11N9	H11	99.41	A/duck/Moscow/6455/2023(H11N9)	PP897307.1
A/Common_teal/Italy/395748_3/2023_HA_H11N9	H11	99.23	A/duck/Moscow/6455/2023(H11N9)	PP897307.1
A/Common_teal/Italy/406997_7/2023_HA_H11N9	H11	98.29	A/duck/Moscow/5712U/2019(H11N6)	MW186787.1
A/Common_teal/Italy/408743_3/2023_HA_H11N9	H11	99.06	A/duck/Moscow/6455/2023(H11N9)	PP897307.1
A/Shoveler/Italy/366521_1/2023_HA_H11N9	H11	99.35	A/duck/Moscow/6455/2023(H11N9)	PP897307.1
A/Shoveler/Italy/366521_2/2023_HA_H11N9	H11	99.35	A/duck/Moscow/6455/2023(H11N9)	PP897307.1
A/Shoveler/Italy/366521_3/2023_HA_H11N9	H11	99.41	A/duck/Moscow/6455/2023(H11N9)	PP897307.1
A/Shoveler/Italy/366521_4/2023_HA_H11N9	H11	99.41	A/duck/Moscow/6455/2023(H11N9)	PP897307.1
A/Shoveler/Italy/366521_5/2023_HA_H11N9	H11	99.29	A/duck/Moscow/6455/2023(H11N9)	PP897307.1
A/Mallard/Italy/376426_1/2023_HA_H11N9	H11	98.41	A/duck/Moscow/5712U/2019(H11N6)	MW186787.1
A/Mallard/Italy/376603_3/2023_HA_H11N9	H11	98.14	A/duck/Moscow/5712U/2019(H11N6)	MW186787.2
A/Wigeon/Italy/378354_3/2023_HA_H11N9	H11	99.35	A/duck/Moscow/6455/2023(H11N9)	PP897307.1
A/Wigeon/Italy/378354_7/2023_HA_H11N9	H11	99.35	A/duck/Moscow/6455/2023(H11N9)	PP897307.1
A/Wigeon/Italy/378354_8/2023_HA_H11N9	H11	99.35	A/duck/Moscow/6455/2023(H11N9)	PP897307.1

A/teal/Italy/414125_10/2022_HA_H12N2	H12	97.46	A/duck/Kaohsiung/20WB0201-52/2020(H12N5)	OR949716.1
A/Common_teal/Italy/337601_5/2022_HA_H12N2	H12	97.28	A/duck/Kaohsiung/20WB0201-52/2020(H12N5)	OR949716.1
A/Common_teal/Italy/337601_1/2022_HA_H12N2	H12	97.28	A/duck/Kaohsiung/20WB0201-52/2020(H12N5)	OR949716.1
A/Northern_pintail/Italy/326377/2022_HA_H12N8	H12	94.50	A/aquatic bird/Jeonju/134/2006(H12N5)	MW547681.1
A/Mallard/Italy/385416/2023_HA_H12N5	H12	97.28	A/duck/Kaohsiung/20WB0201-52/2020(H12N5)	OR949716.1

Table S3. Blast analysis of NA genes for all non-H5 samples.

**Sample**	**NA**	**% identity**	**Closest sequenze**	**Accession no.**
A/Mallard/Italy/23013/2022_NA_H1N1	N1	98.44	A/Anas platyrhynchos/Belgium/10402_H195386/2017(H1N1)	MT439899.1
A/Shoveler/Italy/23061/2022_NA_H1N1	N1	98.65	A/Anas platyrhynchos/Belgium/10402_H195386/2017(H1N1)	MT439899.1
A/Teal/Italy/413742/2022_NA_H6N1	N1	98.72	A/garganey/Egypt/DT20869C/2022(H6N1)	OR786182.1
A/Wigeon/Italy/367477_2/2022_NA_H1N1	N1	98.58	A/Anas platyrhynchos/Belgium/10402_H195386/2017(H1N1)	MT439899.1
A/Wigeon/Italy/367477_3/2022_NA_H1N1	N1	98.58	A/Anas platyrhynchos/Belgium/10402_H195386/2017(H1N1)	MT439899.1
A/Mallard/Italy/390956_2/2022_NA_H6N1	N1	99.22	A/garganey/Egypt/DT20869C/2022(H6N1)	OR786182.1
A/Mallard/Italy/390906_1/2022_NA_H6N1	N1	99.00	A/garganey/Egypt/DT20869C/2022(H6N1)	OR786182.1
A/Mallard/Italy/390906_3/2022_NA_H6N1	N1	99.00	A/garganey/Egypt/DT20869C/2022(H6N1)	OR786182.1
A/Northern_shoveler/Italy/390877_2/2022_NA_H6N1	N1	98.65	A/Anas platyrhynchos/Belgium/1837_H101620/2018(H10N1)	MT406947.1
A/Northern_shoveler/Italy/390877_3/2022_NA_H6N1	N1	98.65	A/Anas platyrhynchos/Belgium/1837_H101620/2018(H10N1)	MT406947.1
A/Common_teal/Italy/390919_8/2022_NA_H6N1	N1	99.99	A/garganey/Egypt/DT20869C/2022(H6N1)	OR786182.1
A/Mallard/Italy/338541_4/2022_NA_H3N1	N1	98.29	A/Anas platyrhynchos/Belgium/10402_H195386/2017(H1N1)	MT439899.1
A/Common_teal/Italy/347389_6/2022_NA_H6N1	N1	99.29	A/garganey/Egypt/DT20869C/2022(H6N1)	OR786182.1
A/Common_teal/Italy/326040_1/2022_NA_H1N1	N1	98.51	A/Anas platyrhynchos/Belgium/10402_H195386/2017(H1N1)	MT439899.1
A/Eurasian_wigeon/Italy/22VIR9645_3/2022_NA_H5N1_Seniga	N1	99.57	A/Cygnus olor/San Nazzaro/V0469_NA/2022(H5N1)	PQ098786.1
A/Mallard/Italy/22VIR9646_1/2022_NA_H5N1_Casalromano	N1	99.79	A/Cygnus olor/San Nazzaro/V0469_NA/2022(H5N1)	PQ098786.1
A/Mallard/Italy/22VIR9909_1/2022_NA_H5N1_Quinzano	N1	99.72	A/Cygnus olor/San Nazzaro/V0469_NA/2022(H5N1)	PQ098786.1
A/Teal/Italy/22VIR11684_2/2022_NA_H5N1_DuomodiRovato	N1	99.72	A/Cygnus olor/Zuerich/V0263/2023(H5N1)	PQ098689.1
A/Teal/Italy/22VIR12727_1/2022_NA_H5N1_DuomodiRovato	N1	99.79	A/Cygnus olor/Zuerich/V0263/2023(H5N1)	PQ098689.1
A/Teal/Italy/22VIR12878/2022_NA_H5N1_DuomodiRovato	N1	99.72	A/Cygnus olor/Zuerich/V0263/2023(H5N1)	PQ098689.1
A/Northern_pintail/Italy/337645/2023_NA_H6N1	N1	99.15	A/garganey/Egypt/DT20869C/2022(H6N1)	OR786182.1
A/Gadwall/Italy/386681_1/2023_NA_H1N1	N1	99.08	A/garganey/Egypt/DT20869C/2022(H6N1)	OR786182.1
A/Gadwall/Italy/386681_2/2023_NA_H1N1	N1	99.08	A/garganey/Egypt/DT20869C/2022(H6N1)	OR786182.1
A/Gadwall/Italy/386681_3/2023_NA_H1N1	N1	99.08	A/garganey/Egypt/DT20869C/2022(H6N1)	OR786182.1
A/Teal/Italy/399256/2022_NA_H6N2	N2	98.93	A/duck/Moscow/5881/2021(H3N2)	OP133626.1
A/Teal/Italy/401707/2022_NA_H6N2	N2	98.79	A/duck/Moscow/5586/2018(H1N2)	MN435637.1
A/Teal/Italy/414125_10/2022_NA_H12N2	N2	98.72	A/duck/Moscow/5881/2021(H3N2)	OP133626.1
A/Mallard/Italy/328086_2/2022_NA_H9N2	N2	98.72	A/duck/Moscow/5586/2018(H1N2)	MN435637.1
A/Mallard/Italy/328091_2/2022_NA_H9N2	N2	98.72	A/duck/Moscow/5586/2018(H1N2)	MN435637.1
A/Common_teal/Italy/337601_5/2022_NA_H12N2	N2	99.21	A/duck/Moscow/5881/2021(H3N2)	OP133626.1
A/Common_teal/Italy/337601_1/2022_NA_H12N2	N2	99.22	A/duck/Moscow/5881/2021(H3N2)	OP133626.1
A/Northern_pintail/Italy/347406_5/2022_NA_H9N2	N2	98.22	A/duck/Moscow/5586/2018(H1N2)	MN435637.1
A/Mallard/Italy/348683_1/2022_NA_H5N2	N2	98.86	A/duck/Moscow/5586/2018(H1N2)	MN435637.1

A/teal/Italy/418637/2022_NA_H9N2	N2	98.86	A/duck/Moscow/5586/2018(H1N2)	MN435637.1
A/Common_teal/Italy/376566_2/2023_NA_H6N2	N2	98.15	A/duck/Moscow/5586/2018(H1N2)	MN435637.1
A/Gadwall/Italy/394037_4/2023_NA_H1N2	N2	98.22	A/duck/Moscow/5586/2018(H1N2)	MN435637.1
A/Shoveler/Italy/394076/2023_NA_H8N2	N2	98.72	A/duck/Moscow/5586/2018(H1N2)	MN435637.1
A/Mallard/Italy/299696_7/2023_NA_H6N2	N2	98.22	A/duck/Moscow/5586/2018(H1N2)	MN435637.1
A/Mallard/Italy/306879_4/2023_NA_H6N2	N2	98.22	A/duck/Moscow/5586/2018(H1N2)	MN435637.1
A/Mallard/Italy/304157_2/2023_NA_HXN2	N2	98.15	A/duck/Moscow/5586/2018(H1N2)	MN435637.1
A/Mallard/Italy/315897_1/2023_NA_H6N2	N2	98.15	A/duck/Moscow/5586/2018(H1N2)	MN435637.1
A/Common_teal/Italy/320322_1/2023_NA_H6N2	N2	98.15	A/duck/Moscow/5586/2018(H1N2)	MN435637.1
A/Common_teal/Italy/342250_2/2023_NA_H6N2	N2	98.65	A/duck/Moscow/5586/2018(H1N2)	MN435637.1
A/Mallard/Italy/342238_2/2023_NA_H6N2	N2	98.15	A/duck/Moscow/5586/2018(H1N2)	MN435637.1
A/Mallard/Italy/355612_1/2022_NA_H2N3	N3	98.72	A/garganey/Egypt/MB-D-1341C/2017(H14N3)	OQ793917.1
A/Mallard/Italy/355612_2/2022_NA_H2N3	N3	98.72	A/garganey/Egypt/MB-D-1341C/2017(H14N3)	OQ793917.1
A/Common_teal/Italy/330815_3/2022_NA_H2N3	N3	98.72	A/garganey/Egypt/MB-D-1341C/2017(H14N3)	OQ793917.1
A/Shoveler/Italy/306797_2/2023_NA_H5N3	N3	99.00	A/mallard/South Korea/JB21-58/2019(H5N3)	MW492929.1
A/Common_teal/Italy/362850_1/2022_NA_H8N4	N4	98.72	A/mallard/Shanghai/JDS120662/2018(H10N4)	MN049528.1
A/Mallard/Italy/385416/2023_NA_H12N5	N5	98.23	A/duck/Cambodia/C70W14M/2018(H7N5)	MN702974.1
A/Mallard/Italy/302598_2/2023_NA_H3N6	N6	97.80	A/duck/Kaohsiung/17WB0127-1/2017(H8N6)	OR949510.1
A/Mallard/Italy/302598_4/2023_NA_H3N6	N6	97.80	A/duck/Kaohsiung/17WB0127-1/2017(H8N6)	OR949510.1
A/Mallard/Italy/321660_2/2023_NA_H4N6	N6	98.09	A/duck/Kaohsiung/17WB0127-1/2017(H8N6)	OR949510.1
A/Common_teal/Italy/386684_3/2023_NA_H4N6	N6	97.30	A/duck/Kaohsiung/17WB0127-1/2017(H8N6)	OR949510.1
A/Mallard/Italy/335123_7/2022_NA_H8N8	N8	98.79	A/northern shoveler/North-Kazakhstan/20/2018(H3N8)	MN945307.1
A/Mallard/Italy/335123_5/2022_NA_H8N8	N8	98.79	A/northern shoveler/North-Kazakhstan/20/2018(H3N8)	MN945307.1
A/Mallard/Italy/321045_3/2022_NA_H3N8	N8	98.94	A/northern shoveler/North-Kazakhstan/20/2018(H3N8)	MN945307.1
A/Mallard/Italy/321045_4/2022_NA_H3N8	N8	98.94	A/northern shoveler/North-Kazakhstan/20/2018(H3N8)	MN945307.1
A/Mallard/Italy/321045_6/2022_NA_H3N8	N8	98.87	A/northern shoveler/North-Kazakhstan/20/2018(H3N8)	MN945307.1
A/Mallard/Italy/321045_7/2022_NA_H3N8	N8	98.94	A/northern shoveler/North-Kazakhstan/20/2018(H3N8)	MN945307.1
A/Mallard/Italy/321045_8/2022_NA_H3N8	N8	98.94	A/northern shoveler/North-Kazakhstan/20/2018(H3N8)	MN945307.1
A/Common_teal/Italy/321099_1/2022_NA_H3N8	N8	98.87	A/northern shoveler/North-Kazakhstan/20/2018(H3N8)	MN945307.1
A/Northern_pintail/Italy/326377/2022_NA_H12N8	N8	99.08	A/duck/Chernogolovka/5897/2021(H3N8)	OP136013.1
A/Common_teal/Italy/326485/2022_NA_H3N8	N8	99.08	A/northern shoveler/North-Kazakhstan/20/2018(H3N8)	MN945307.1
A/Mallard/Italy/321045_1/2022_NA_H3N8	N8	98.94	A/northern shoveler/North-Kazakhstan/20/2018(H3N8)	MN945307.1
A/Mallard/Italy/321045_2/2022_NA_H3N8	N8	98.94	A/northern shoveler/North-Kazakhstan/20/2018(H3N8)	MN945307.1
A/Mallard/Italy/321045_5/2022_NA_H3N8	N8	98.82	A/northern shoveler/North-Kazakhstan/20/2018(H3N8)	MN945307.1
A/Mallard/Italy/379789_2/2023_NA_H6N8	N8	98.80	A/Anas platyrhynchos/Belgium/7827/2018(H3N8)	MT407035.1
A/Shoveler/Italy/392262_2/2023_NA_H6N8	N8	98.37	A/duck/Chernogolovka/5897/2021(H3N8)	OP136013.1
A/Shoveler/Italy/392262_3/2023_NA_H6N8	N8	98.37	A/duck/Chernogolovka/5897/2021(H3N8)	OP136013.1
A/Common_teal/Italy/407012_2/2023_NA_H6N8	N8	98.44	A/Anas platyrhynchos/Belgium/7827/2018(H3N8)	MT407035.1
A/Mallard/Italy/407002_2/2023_NA_H6N8	N8	98.44	A/Anas platyrhynchos/Belgium/7827/2018(H3N8)	MT407035.1
A/Mallard/Italy/407002_3/2023_NA_H6N8	N8	98.44	A/Anas platyrhynchos/Belgium/7827/2018(H3N8)	MT407035.1
A/Common_teal/Italy/407012_5/2023_NA_H6N8	N8	98.44	A/Anas platyrhynchos/Belgium/7827/2018(H3N8)	MT407035.1
A/Common_teal/Italy/407012_4/2023_NA_H6N8	N8	98.79	A/Anas platyrhynchos/Belgium/7827/2018(H3N8)	MT407035.1

A/Mallard/Italy/304157_1/2023_NA_H3N8	N8	99.02	A/duck/Chernogolovka/5897/2021(H3N8)	OP136013.1
A/Mallard/Italy/299740_5/2023_NA_H3N8	N8	98.51	A/duck/Chernogolovka/5897/2021(H3N8)	OP136013.1
A/Mallard/Italy/321660_1/2023_NA_H3N8	N8	98.94	A/Arenaria interpres/Belgium/9365_2/2019(H3N8)	MT407147.1
A/Mallard/Italy/321667_2/2023_NA_H3N8	N8	98.94	A/Arenaria interpres/Belgium/9365_2/2019(H3N8)	MT407147.1
A/Common_teal/Italy/336752_4/2023_NA_H3N8	N8	98.44	A/duck/Chernogolovka/5897/2021(H3N8)	OP136013.1
A/Wigeon/Italy/336772_1/2023_NA_H3N8	N8	98.44	A/duck/Chernogolovka/5897/2021(H3N8)	OP136013.1
A/Wigeon/Italy/336772_2/2023_NA_H3N8	N8	98.44	A/duck/Chernogolovka/5897/2021(H3N8)	OP136013.1
A/Teal/Italy/417152_1/2022_NA_H11N9	N9	97.94	A/teal/Egypt/MB-D-621C/2016(H7N9)	MN208027.1
A/Teal/Italy/422038_2/2022_NA_H11N9	N9	96.95	A/ibis/Zambia/1765/2015(H2N9)	OQ120637.1
A/Mallard/Italy/432522_1/2022_NA_H11N9	N9	97.12	A/ibis/Zambia/1765/2015(H2N9)	OQ120637.1
A/Mallard/Italy/4854_1/2023_NA_H11N9	N9	97.16	A/ibis/Zambia/1765/2015(H2N9)	OQ120637.1
A/Mallard/Italy/359760/2022_NA_H11N9	N9	97.38	A/ibis/Zambia/1765/2015(H2N9)	OQ120637.1
A/Wigeon/Italy/378354_3/2023_NA_H11N9	N9	99.36	A/duck/Moscow/6454/2023(H11N9)	PP897299.1
A/Wigeon/Italy/378354_7/2023_NA_H11N9	N9	99.36	A/duck/Moscow/6454/2023(H11N9)	PP897299.1
A/Wigeon/Italy/378354_8/2023_NA_H11N9	N9	99.36	A/duck/Moscow/6454/2023(H11N9)	PP897299.1
A/Common_teal/Italy/395613_5/2023_NA_H11N9	N9	99.37	A/duck/Moscow/6454/2023(H11N9)	PP897299.1
A/Common_teal/Italy/395748_3/2023_NA_H11N9	N9	99.36	A/duck/Moscow/6454/2023(H11N9)	PP897299.1
A/Common_teal/Italy/406997_7/2023_NA_H11N9	N9	97.23	A/ibis/Zambia/1765/2015(H2N9)	OQ120637.1
A/Common_teal/Italy/408743_3/2023_NA_H11N9	N9	99.29	A/duck/Moscow/6454/2023(H11N9)	PP897299.1
A/Mallard/Italy/386690_2/2023_NA_H11N9	N9	99.25	A/duck/Moscow/6454/2023(H11N9)	PP897299.1
A/Shoveler/Italy/366521_1/2023_NA_H11N9	N9	99.26	A/duck/Moscow/6454/2023(H11N9)	PP897299.1
A/Shoveler/Italy/366521_2/2023_NA_H11N9	N9	99.26	A/duck/Moscow/6454/2023(H11N9)	PP897299.1
A/Shoveler/Italy/366521_3/2023_NA_H11N9	N9	99.29	A/duck/Moscow/6454/2023(H11N9)	PP897299.1
A/Shoveler/Italy/366521_4/2023_NA_H11N9	N9	99.29	A/duck/Moscow/6454/2023(H11N9)	PP897299.1
A/Shoveler/Italy/366521_5/2023_NA_H11N9	N9	99.29	A/duck/Moscow/6454/2023(H11N9)	PP897299.1
A/Mallard/Italy/376603_3/2023_NA_H11N9	N9	97.45	A/ibis/Zambia/1765/2015(H2N9)	OQ120637.1
A/Mallard/Italy/376426_1/2023_NA_H11N9	N9	97.23	A/ibis/Zambia/1765/2015(H2N9)	OQ120637.1
A/Gadwall/Italy/386681_4/2023_NA_H11N9	N9	99.29	A/duck/Moscow/6454/2023(H11N9)	PP897299.1
